# Supplementary material for: Analog optical computer for AI inference and combinatorial optimization
Source: Nature. 2025 Sep 3;645(8080):354–61. doi: 10.1038/s41586-025-09430-z (PMC12422976; doi:10.1038/s41586-025-09430-z)
Supplement: Supplementary file 1 — This file contains sections A–G, including Supplementary Figs. 1–13, 4 Supplementary Tables and References. [file 41586_2025_9430_MOESM1_ESM.pdf]

nature portfolio

https://doi.org/10.1038/s41586-025-09430-z

Supplementary information

Analog optical computer for AI inference and combinatorial optimization

In the format provided by the authors and unedited

Supplementary Materials

Kirill P. Kalinin[1*], Jannes Gladrow[1*], Jiaqi Chu[1], James H. Clegg[1], Daniel Cletheroe[1], Doug J. Kelly[1], Babak Rahmani[1], Grace Brennan[1], Burcu Canakci[1], Fabian Falck[1], Michael Hansen[2], Jim Kleewein[2], Heiner Kremer[1], Greg O'Shea[1], Lucinda Pickup[1], Saravan Rajmohan[2], Ant Rowstron[1], Victor Ruhle[2], Lee Braine[3], Shrirang Khedekar[3], Natalia G. Berloff[1,4], Christos Gkantsidis[1], Francesca Parmigiani[1*], Hitesh Ballani[1*]

[1] *Microsoft Research, Cambridge, UK*

[2] *Microsoft, Redmond, WA, USA*

[3] *Chief Technology Office, Barclays, London, UK*

[4] *Department of Applied Mathematics and Theoretical Physics, University of Cambridge, UK*

[*] kkalinin@microsoft.com jannes.gladrow@microsoft.com francesca.parmigiani@microsoft.com hitesh.ballani@microsoft.com

Table of Contents

A *AOC* hardware at scale **4**

A.1 Scaling, computing speed, and energy estimations 4

B AOC hardware implementation **9**

B.1 Optical subsystem for matrix-vector multiplication 9

B.2 Analog electronic subsystem for nonlinear, annealing, summing, and differencing operations 14

B.3 Evaluation of dot-product and matrix-vector multiplication accuracies 18

C Analog equilibrium models **20**

C.1 Additional classification results 20

D Non-idealities and their impact on ML performance **26**

D.1 Signal-to-noise ratio (SNR) 27

D.2 Non-ideality Details 28

E Repeated runs and averaging **31**

F Increased Out-of-Distribution Generalization and Equilibrium Model Robustness **32**

F.1 Addition Task 33

F.2 PrefixSum Task 34

F.3 Maze solving task 35

F.4 Noise Robustness 35

G *AOC* for optimization **37**

G.1 Algorithmic approach for optimization 37

G.2 *AOC* hardware performance in optimization 40

G.3 Medical image reconstruction 45

G.4 *AOC* parameters for optimization 48

G.5 *AOC-DT* performance at scale in optimization 48

G.6 Competing solvers in optimization benchmarks 54

G.7 Optimization benchmarks description 56

G.8 Problem mapping advantage of *QUMO* abstraction over *QUBO* model 58

G.9 Quantum hardware limitations for optimization problems 62

G.10 Physical analogy of the *AOC* algorithmic approach in optimization 64

G.11 Comparison of the *AOC* approach to other iterative approaches . . 67

References **71**

A *AOC* hardware at scale

A.1 Scaling, computing speed, and energy estimations

In what follows we consider how to scale an *AOC* system to a large number of total weights N_{tot}, for example $0.1 - 2\text{billion}$, and estimate the computing efficiency of the system. N, the number of weights in an individual module, will be limited, for example to $4\ \text{million}$. *AOC* will be composed of multiple modules connected together. Multiplication of a large vector and matrix can be achieved by partitioning the vector and matrix to smaller blocks, performing matrix-vector multiplication across these blocks, and then summing the partial results. We envision that each module would perform the matrix-vector multiplication corresponding to one block. Multiple modules of identical performance would be connected together with the inter-block summation happening in analog electronics. The number of modules in *AOC* is determined only by the total required weights and the number of weights of per module.

Optical and electronic integration is essential to the scaling effort. On the optical side, the scaling will require miniaturising current 3D optics through arrays of micro-lenses, possibly in combination with 3D waveguides for fan-in and fan-out, aiming for an overall path length of a few centimeters per module. While further investigation is needed to confirm such design feasibility at scale, recent advancements in laser-written waveguide devices[1] and high-precision 3D printing

via two-photon polymerization[2] provide promising directions. We propose that each microLED has an active area of $5\ \mu$m and that the array pitch is $10\ \mu$m, to maintain the same ratio as the current implementation. This results in a die size of 2 cm for 2000 variables. To achieve sufficient signal-to-noise ratios and dynamic ranges, each variable is represented by multiple dependent microLEDs, with their output captured by multiple dependent photodetectors. For example, 20 microLEDs can be used to represent each variable or activation with each microLED fanning-out to 100 weights. The small size of the microLEDs allows their operation bandwidth to reach our 2 GHz target.

Our architecture efficiently transitions between optical and analog electronic domains at each iteration, where nonlinearity and annealing are implemented. Optical-to-electronic conversions and vice versa are performed using Si-photodetectors and microLEDs, eliminating the energy overhead of repeated analog-to-digital and digital-to-analog conversions. The in-memory design ensures that signals remain in the analog domain until convergence, mitigating data movement constraints.

An important factor in scalability is our choice of surface-emitting sources and photodetectors, which are crucial for leveraging the third dimension, where the computation is happening. MicroLEDs and Si Photodetectors can be grown on silicon, enabling tight integration with an electronic backplane to support large arrays.

These individual modules are connected together in a 3D mesh structure (see Extended Data Fig. 4) to minimize the overall size of the system and consequently

the round-trip time (iteration time) of the system. Additionally, summation of per-module results and dissemination of signals between modules can happen across a predetermined subgroup of modules. We can reduce the round-trip time further by laying modules in such subgroups as close to each other as possible. We note the difference in delays among modules needs to be below one iteration time to avoid the need for an analog clock to synchronize modules.

AOC operates in the visible range, allowing us to use Silicon photodetectors or CMOS sensors. This brings two advantages: first, we can use well-established and reliable CMOS ecosystem and technology, leading to reduced costs; second, because it shares the same CMOS technology as the analog electronics, we envisage a monolithic design where the PD array and the analog electronic components are on a single silicon die.

The computing speed $\mathcal{C}$, measured in operations per second (OPS), of *AOC* at scale is calculated as the number of multiplications and additions, N_{MAC}, in each iteration time, T_{frame}. Since both multiplications and additions scale with the number of weights and $N_{\text{tot}} >> 1$ we approximate $N_{\text{MAC}} = 2N_{\text{tot}}$. T_{frame} is primarily limited by the optoelectronic component bandwidth, $\mathcal{H} = 2$ GHz. Therefore $\mathcal{C} = \mathcal{H}N_{\text{MAC}}$. With $N_{\text{tot}} = 1 \times 10^8$ (25 modules each with 4×10^6 weights) we have a computational speed $\mathcal{C} = 0.4$ EOPS.

To estimate the power consumption of *AOC*, we account for the entire system architecture, including optics, electronics, analog-to-digital conversion (ADC) and

digital-to-analog conversion (DAC), as well as memory read/write operations. Since the SLMs, where weights are stored, have refresh rates much slower than the *AOC*, their operating bandwidth is low and so their contribution to the overall system power is negligible. Similar considerations are applied to the power consumption of the read out and corresponding ADC: the system iterates tens to thousands of times before finding the fixed points and the corresponding results area read out at sample rates of 200 MHz or lower which is more than an order of magnitude slower than the iteration speed. For reference, in,[3] it is reported a power consumption of 133 uW at 150 MS/s 7 bit ADC in 90 nm digital CMOS, accounting for only 1 W of power at the *AOC* system scale. Therefore, the primary power contributors are the modulation of optical sources, P_{source}, and post-detection amplification through TIAs, P_{TIA}. We consider a power consumption of 2.2 mW per TIA[4] with a fan-in ratio of $667:1$ per module (or 3 TIAs per module per sign), so the total power contribution coming from the TIAs is 659.7 W

The estimation of P_{source} is more complex. The calculation of the required received optical power for the system to run accurately within the chosen precision involves two key objectives: i. reading out results with 8 bit precision at 100 MHz, and ii. supporting analog computations at 2 GHz as the system converges. The first point implies an optical received power of about -11 dBm, while the second one requires about -22 dBm, respectively. So, taking into account the worse case between the two, assuming a 30% optical conversion efficiency, and a system loss of 10 dB, the required total power for the optical sources is about 146.8 W.

Therefore, for 100 million weights, the total estimated power for *AOC* is in the order of 806.5 W, resulting in a system efficiency of about 495 TOPS/W. Here we have included the power consumption for 50 modules with $N_{\text{tot}} = 1 \times 10^8$ and 4×10^6 weights per module to allow for the possibility of performing positive and negative multiplications on different modules.[5] Much higher system efficiencies can be envisioned by using a single SLM for positive and negative calculations (and trading 1 bit precision of the SLM); by increasing the fan-in ratios; or by increasing the system bandwidth. We note that these potential speed and efficiency gains come from exploiting the trade-off between generality and efficiency that is fundamental in hardware design. *AOC* explores this trade-off by specializing the hardware for the specific tasks of optimization and machine learning and pushing it further in the context of opto-analog hardware.

B AOC hardware implementation

B.1 Optical subsystem for matrix-vector multiplication

A photo of *AOC*, used to simultaneously run ML models and optimization instances, is shown in Extended Data Fig. 1a, with the highlighted key components. A photo of *AOC* inside a rack, with all the equipment needed in shown in Extended Data Fig. 1b. The OVMM setup, whose schematic diagram is shown in Extended Data Fig. 2, consists of an array of light sources, two SLMs, two arrays of photodetectors and an imaging system. The high level operation is described in the Methods section.

Our microLED array is custom-fabricated. The microLEDs are structurally identical to those used for display applications except that we can control each source individually. An image of the 1×16 green microLEDs taken under an inspection microscope and imaged onto a camera is shown in Supplementary Fig. 1a and Supplementary Fig. 1b. Undesired reflections from the sidewall of the mesa are visible. This may lead to increased inter-variable crosstalk. This can be mitigated in future fabrication runs with a more optimized geometry. Supplementary Fig. 1c shows the microLED emission spectra. The LEDs have a -3 dB bandwidth of 60 MHz at 5 mA, 200 MHz at 20 mA and 350 MHz at 40 mA, respectively, Supplementary Fig. 1d. The normalized optical powers of the 16 microLEDs as a function of the driving voltage are shown in Supplementary Fig. 1e. This shows that there is some nonlinearity as a function of the driving current and also that there is some variation

in the optical power between the sources.

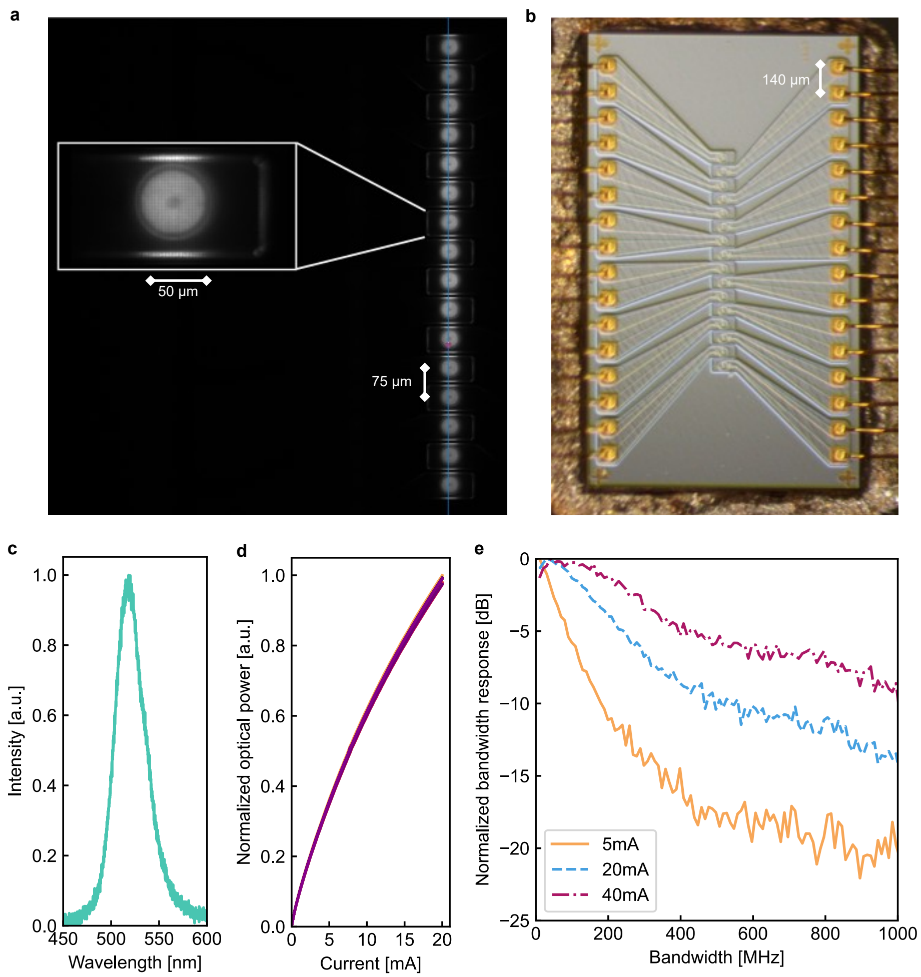

Supplementary Fig. 1: Key properties of microLEDs. **1×16 microLEDs imaged onto a camera (a) and taken under an inspection microscope (b). The size of each microLED is 50 μm in diameter, with a pitch of 75 μm. The size of the electric pads are $90\,\mu$m $\times$ $90\,\mu$m, a pitch of 140 μm and a gap of 50 μm. (c) Example of a measured microLED emission spectrum. (d) Normalized measurements of the 16 microLEDs optical powers as a function of their driving current. (e) Examples of measured microLED bandwidth responses at 5 mA, 20 mA and 40 mA, respectively.**

To generate polarized light from the unpolarized microLED output we use a polarizing beamsplitter (PBS) (see Extended Data Fig. 2). We use an input linear polarization at 45 degrees to the director of the liquid crystal (horizontal polarization for one SLM and vertical for the other) so that we can modulate the polarization of the output light. The light then becomes amplitude-modulated after the backwards pass through the first PBS.

The second PBS in Extended Data Fig. 2 splits the signals originating from the non-negative and non-positive multiplications and directs them to the correct PD arrays. A close up image of one of the two SLMs is shown in Supplementary Fig. 2a. The 16 fanned-out beam profiles are shown in Supplementary Fig. 2b. The microLED pitch at the SLM is 293 μm. The SLM has 8.5 μm pixel pitch and a fill factor of 92%. Supplementary Fig. 2.c shows an example of the modulated intensity as a function of the grey value after the look up table (LUT) is applied.

Each of the two PD arrays contains 48 rectangular PDs (though, we only use 16 PDs per multiplication sign in the work presented here). The PD arrays were custom fabricated to our specifications by an external partner.

Novel optics for fan-in and fan-out to implement matrix-vector multiplication

In order to implement our OVMM setup we designed a novel free space optical system that combines spherical and cylindrical optical elements (see Extended Data Fig. 2). The Stanford optical vector by matrix multiplication design uses an interleaved 4F

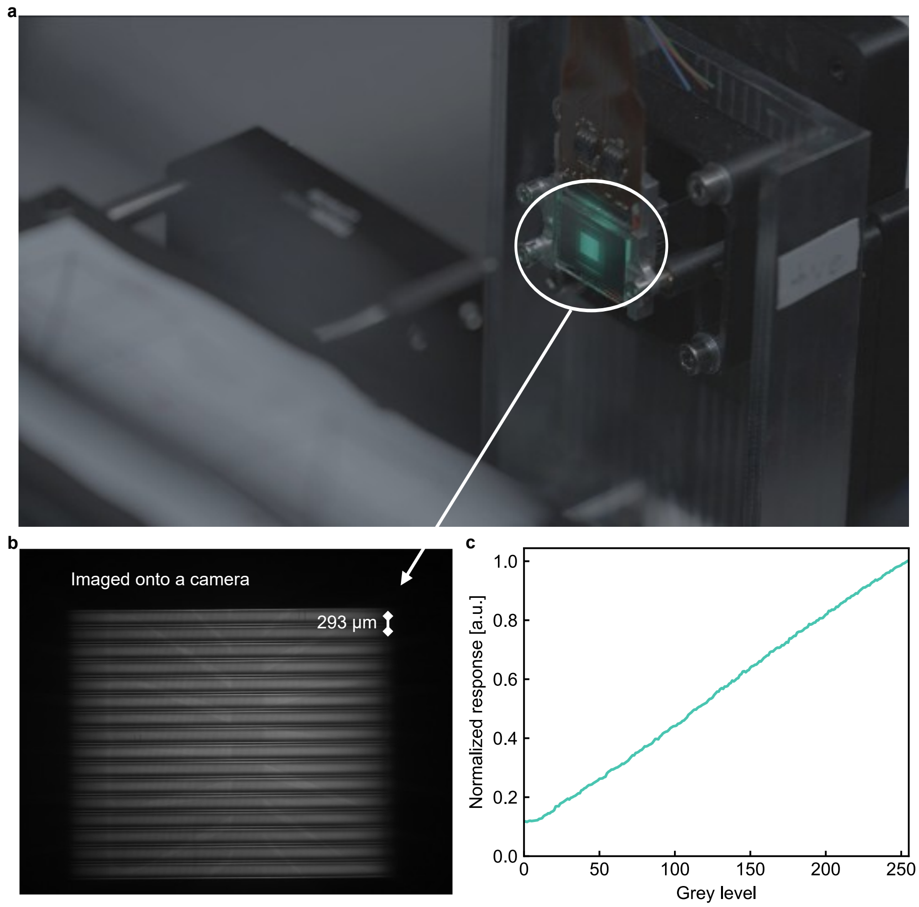

Supplementary Fig. 2: Key properties of SLMs. (a) Close up photo of one SLM. (b) 16 fanned-out beam profiles shining on one SLM and imaged onto a camera. The microLED pitch at the SLM is 293 μm and the SLM has 8.5 μm pixel pitch and a fill factor of 92%. (c) Normalized SLM transfer function over the grey values.

and 2F system with cylindrical lenses.[6–8] The 4F system typically images the sources on onto the modulator (matrix) and the 2F system performs the Fourier transform in the orthogonal spatial direction. There are a number challenges with this approach. First, it is hard to make high quality (i.e., small RMS spot size) prototype optical designs because cylindrical lenses are only readily available in plano-form. Second, it

is hard to make high efficiency optical designs for divergent sources. This is because the lenses forming the 2F system, which perform the spreading operation, must be as close to the source as possible, which means they must have large NA and therefore be very powerful. Now, the lenses in the 4F system must be then even closer to the sources than the 2F lenses, which means that the optical path becomes packed with solid material (e.g., glass) and it is hard to include other components, for example beamsplitters. Finally, cylindrical lenses are expensive to manufacture. There is an additional benefit to our 4F-like architecture, which is that in such optical systems, the imaging operation equalises the path length to within the aberration of the optical system, typically a few optical wavelengths. This approach significantly minimizes the impact of propagation-induced time delays for different optical paths, preserving processing accuracy and speed.

In order to overcome the challenges of the Stanford approach we decided to make best use of spherical optics and rely on the minimum possible cylindrical optical power. Spherical optics with high quality and high collection efficiency, for example microscope objectives, are readily available off the shelf. To create the required spreading of each microLED source over a row of the matrix we introduce a relatively weak cylindrical lens in infinity space. This causes the image to appear out-of-focus on the modulator, but only in one direction.

The microscope objective is a Thorlabs TL10X-2P, which has an NA of 0.5 and a field of view of 2.2 mm. We need the high NA in order to collect sufficient light (approximately 25% in this case as the sources are Lambertian) and the large field of

view to image all 16 sources. The design requirements for the second lens group in the 4F system (LG1 in Extended Data Fig. 1d) are more relaxed because the microLED separation is much greater than the diffraction limit. We use two achromatic doublets: Thorlabs AC508–100-A-ML and AC508–300-A-ML. We optimized the choice and orientation of the doublets using Zemax so that each source could be resolved on the SLM with best separation.

The fan-in operation is performed by projecting light onto a set of rectangular PDs. The projection optics is a 4F system composed of two lens groups, LG1 and LG2 in Extended Data Fig. 1d, each with two achromatic doublets, to image the SLM onto the PD array. LG2 is made of a Thorlabs AC508–150-A-ML and a Thorlabs AC508–080-A-ML.

B.2 Analog electronic subsystem for nonlinear, annealing, summing, and differencing operations

The custom electronics uses off-the-shelf surface mount components on PCBs (see Extended Data Fig. 1a). This allows for rapid prototyping of the system at the cost of increased size and power consumption. *AOC* at scale will use analogue ASICs to mitigate these issues. The electronics are divided into three types of PCBs containing i. microLED and driver amplifiers (called microLED board), ii. analogue processing per channel (called main board), and iii. PD and TIA (called PD board). Their

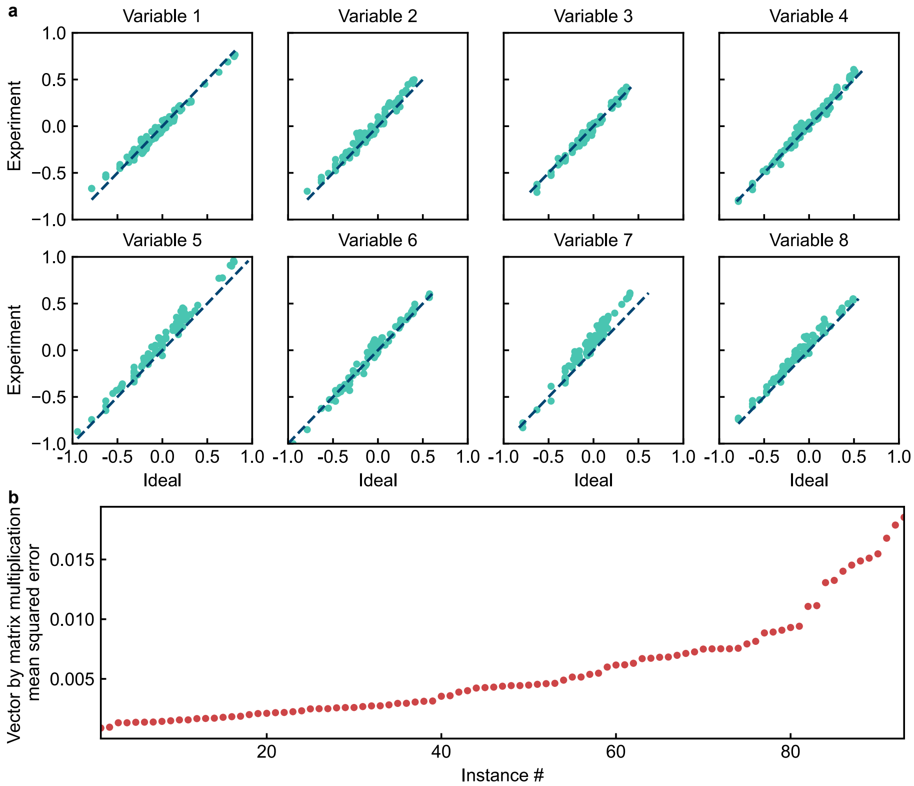

Supplementary Fig. 3: Accuracy study (a) Examples of experimental vs ideal dot-products for 8 variables using 93 reference matrices loaded on each SLM. The same input across all the channels is injected as an AC signal at the test port of the main board. This is a rectangular waveform with amplitude of 0.4 V (b) OVMM MSEs for each instance.

corresponding schematic, including the optical part, is shown in Extended Data Fig. 3. These boards are interconnected through multichannel micro-coax cables, which facilitate ease of prototyping on the optical bench (albeit with some additional delay).

Main analogue board The main board provides the majority of the analogue electronic processing, see Extended Data Fig. 1a. It includes the key analog building block, namely the per-channel nonlinear operation. It also includes debug and test ports, interface to computer for control of on-board VGAs via I2C, and connections for injection of test signals and annealing and momentum waveforms. Extended Data Fig. 3 shows that the annealing path is implemented in the electrical domain as an additional loop after the switch. The annealing signal rejoins the loop at the common VGA. The gain of this path is controlled with another VGA which receives its control voltage from an external analogue input port. This control signal is synthesized from an arbitrary signal generator that can produce any annealing schedule. We typically use a linear ramp which ramps down to $\alpha = 0$ after about 35% of the solve process. The annealing signal is synchronized with the main loop switch control to retrigger the schedule at the beginning of each solve. For annealing, all channels receive the same external control signal. There is also a per-channel offset to allow minor adjustment or correction of channel-to-channel variations. The momentum path is implemented in a similar way to the annealing path. As with the momentum, the output after the switch is fed back through a VGA and rejoins at the common VGA. This loop differs in that the time derivative is performed in the electrical domain using the VGA with an additional capacitor to form a differentiator circuit. In future work, we plan to expand the operation capabilities of this module to include normalization, softmax, and additional functions that are relevant for optimisation and *ML* workloads and can be implemented efficiently in the analog domain.

For the machine learning workload the momentum circuit is repurposed as an injection port. Here we inject a vector that is constant for the duration of solve but programable depending on the problem. To achieve this, the loop is broken and, instead of feeding back the derivative, the output of a per channel DAC is passed through the VGA. As this is connected to one of the summing inputs of the common amplifier, these signals are added into to the signal chain at this point.

Alternative ways to realize nonlinearity in optical domain Over the years, numerous impressive efforts have been made to achieve efficient optical nonlinearities, see reference[9] and additional citations within this paper. However, these approaches typically require high peak-power optical pumps, which are often incompatible with low-power, scalable implementations. Similarly, while all-optical loop architectures have been successfully demonstrated,[10] they generally rely on coherent signals for feedback, imposing stringent precision requirements that are in the order of the signal wavelength (hundreds of THz). On the other hand, by leveraging incoherent optical sources, amenable to wafer-scale manufacturing, our architecture, based on incoherent signals, requires only GHz-level optical path matching, potentially enabling a more scalable and efficient *AOC* system.

microLED board The microLED board is separated into two parts to drive the sources: i. a current source is used to set the operating (bias) point; ii. a power amplifier is used to superimpose the signal from the output of the analogue chain. The

bias point current sets the optical power that represents the value zero. The power amplifier is then used to create negative (positive) values by reducing (increasing) the power relative to the bias point. Each microLED has a nonlinear behavior of optical power as a function of drive current (see Supplementary Fig. 1d). We can control this nonlinearity to some extent by selecting the source impedance of the driver. This resistance is added in series with the low output impedance of the power amplifier. This resistance does not affect the current source as it has high impedance. We found $25\ \Omega$ to be optimal by extracting the current/voltage and current/optical power curves and modelling.

Photodiode board There are two PD PCBs inputs per channel: one for non-negative multiplications and the other for non-positive multiplications. The PCB is mounted to the PD chip by wire-bonding and contains linear TIAs with differential outputs.

B.3 Evaluation of dot-product and matrix-vector multiplication accuracies

We describe the characterization of OVMM accuracy. Given an instance, we calculate digitally the correct output of the vector-by-matrix multiplication for each variable. We upload these matrices one-by-one onto the SLM and measure the output of the system and plot the correlation between the measured and ideal values in

Supplementary Fig. 3.a. Here we only show results for 8 channels but results for all 16 show the same behavior. We set all the gains as they would be set in the typical operation mode of the closed loop solver. We measure the signal at the output of one loop (ADC output of the main board). We use a Picoscope with a sample rate of 6.25 MHz and capture 100 samples during the on-time. To evaluate the numerical result we average over 60 samples from sample number 20 to 79 inclusive to avoid transients. The results are then normalized such that the answers for all the used dot products fall between -1 and 1 and are plotted in Supplementary Fig. 3a. The mean MSE across all dot-products is 5.5×10^{-3}, and the VMM MSE as a function of matrix (instance) is shown in Supplementary Fig. 3b, where we ordered the instances from lowest to highest MSE.

C Analog equilibrium models

C.1 Additional classification results

Here we provide additional results obtained on the full MNIST test dataset that allow for a comparison between equilibrium models run on AOC against standard feedforward models as well as models run on the digital twin (DT). In Supplementary Fig. 4a, we provide an extensive comparison. For models with 256 weights in the central weight matrix, we find that AOC-256 (left green bar), AOC-DT-256 (left red bar), as well as ideal DEQ models (left grey bar) behave similarly. Feedforward models with a similar structure, that is, a linear input and output projection sandwiching a single 16×16-layer with a $\tanh$ activation function, perform slightly worse (left dark-grey bar).

For larger models with 4096 weights in the central section (either equilibrium or feedforward), we find markedly improved performance for the AOC-4096 and AOC-DT-4096 models. Interestingly, increasing the parameter count further only gives marginal improvements as can be seen from the red AOC-DT-262k and grey DEQ-262k bars. The ideal DEQ here appears to perform a marginal amount worse which might be caused by the *AOC-DT* models making use of the additional nonlinearities created by the hardware non-idealities (see Appendix D). Again, we find that a pure feedforward model (dark grey bar) in the 262k-weight class performs slightly worse, indicating that a single nonlinearity is insufficient.

Only by adding convolutional operations to the equilibrium model does the performance improve significantly (light grey bar on the right). The convolutional model has a residual block architecture with 64 3×3-convolutional filters and a $\tanh$ activation function. The input and output projections in this case do have nonlinearities and are thus no longer pure projections: the input model consists of a 2D convolution with 64 filters and a 2D max-pooling layer, followed by a ReLU, and the output model consists of three MLPs going from $3136 \rightarrow 200$ dimensions, $200 \rightarrow 200$, and $200 \rightarrow 10$ with ReLU activations, respectively. We added this model (right-most bar) to show that the equilibrium model paradigm can, in principle, offer strong performance, reaching 99% accuracy on MNIST.

In table 2, we summarize the results obtained for the regression tasks.

MNIST results shown in Fig. 2d are summarised in Supplementary 4 again for clarity.

Confusion matrices. In Supplementary Fig. 4b, we plot the confusion matrices obtained for the AOC-256 and AOC-DT-256 models for MNIST. We find the confusion matrices are concentrated along the diagonal. Interestingly, the *AOC*-256 performs worst for digit class 3.

Random input projections. While a larger *AOC* machine in the future could carry out the input (and output) projection in the analog domain, this currently

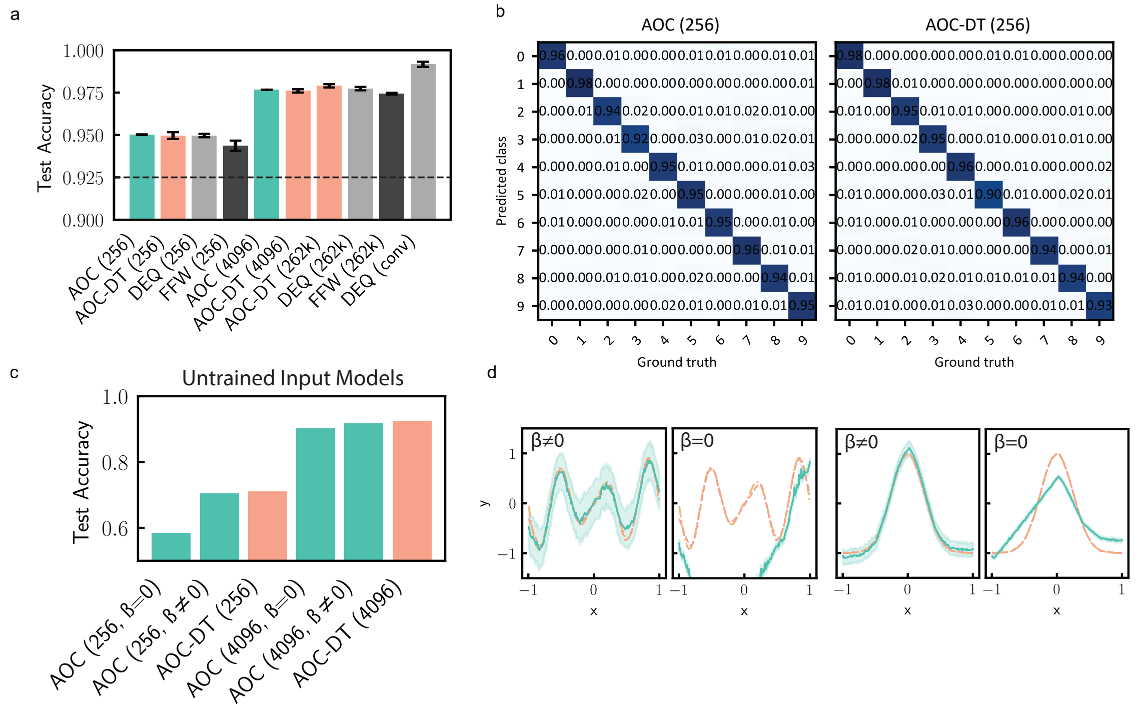

Supplementary Fig. 4: Additional results for the equilibrium model. a, Comparison of equilibrium model results obtained on hardware (green bars), using the digital twin (red bars), or using ideal digital models (light grey) with results obtained on standard feedforward models (dark grey). The performance of a linear classifier is indicated by the dashed line. b, Confusion matrices obtained for the AOC-256 and AOC-DT-256 MNIST classification results shown in panel a and Fig. 2d. c, MNIST classification results obtained when the input projection is and remains randomly initialized. Here, we compare results obtained with $\beta = 0$ (no optical weights) with β at its standard value. The difference gives an indication of the contribution of the 256-optical-weight matrix. d, Contribution of the optical weights for regression tasks. We compare $\beta = 0$ and β at its standard value for the Gaussian and Sinusoidal regression tasks.

would require optical weight matrices with sizes upwards of $10,000$ weights. For now, we have to carry out these projections digitally. The drawback of this, however, is that the input-projection matrix ($784 \times 16 = 12\,544$) can dominate the model

Dataset	Hardware Loss (MSE)	DT Loss (MSE)
Gaussian	3.75e-3	3.83e-3
Sinusoidal	1.21e-2	7.05e-3

Supplementary Table 2: Regression task results obtained on the AOC hardware and DT.

Model	MNIST Accuracy (%)	FashionMNIST Accuracy (%)
AOC-256	95.02 ± 0.02	86.04 ± 0.00
AOC-DT-256	94.97 ± 0.40	86.20 ± 0.80
FFW-256	94.37 ± 0.60	85.63 ± 0.40
AOC-4096	97.67 ± 0.00	89.01 ± 0.00
AOC-DT-4096	97.60 ± 0.20	88.70 ± 0.50
FFW-4096	96.53 ± 0.10	87.43 ± 0.10
Linear Classifier	92.5	84.5

Supplementary Table 4: MNIST classification results obtained on the AOC hardware and DT as well as using standard single-layer feedforward networks with a tanh-nonlinearity and linear input and output projections. Error estimates for digital runs indicate the span of results achieved over 3 different random seeds. For analog runs, the error estimation is described in the methods section.

performance. Already the linear classifier (dashed line in Supplementary Fig. 4a) reaches an accuracy of around $92.5\,\%$ on MNIST.

This means that the features being injected through $\mathbf{x}_{\text{proj}}$ in equation 2 may already be informative. The main contribution of our machine in such cases is to provide a nonlinearity. Indeed, augmenting this linear classifier with a single $\tanh$ nonlinearity in the central section of the model, which effectively turns it into a feedforward neural network, allows it to reach 94.37% on MNIST (see Supplementary Fig. 4a, leftmost dark grey bar).

We therefore conduct an ablation study where we leave the input projection untrained and frozen during training of the model on the digital twin. The output projection, however, is still being trained since its size $(16 \times 10 = 160)$ is sufficiently small. We find that applying a selu activation to the random input projection renders its features incrementally more informative.

With these untrained, random input features, AOC-256 obtains an MNIST test accuracy of $70.47\,\%$ (see second bar from left in Supplementary Fig. 4c), while AOC-4096 obtains $91.74\,\%$. We find that for random input features, the *AOC-DT* struggles to accurately model the hardware. The fidelity decreases to 88% of labels for AOC-256 and 98% for AOC-4096. We observe that inputs to the machine have much smaller differences which are challenging to resolve.

For random input features, the optical weights have a chance to contribute meaningfully to the result. We can get an impression of their contribution by switching the β gain to zero, which removes the weight matrix from the equation. For $\beta = 0$, the accuracy drops to $53.39\,\%$ for AOC-256 and $90.02\,\%$ for AOC-4096. The smaller drop for the larger model is likely due to the larger output projection which has to grow with the size of the central equilibrium model.

We can repeat a similar study for the regression tasks. For regression tasks, the projections are of a lesser concern since they each only have 16-matrix-weight parameters. We therefore leave the input and output projections linear, and trained and study the impact of setting $\beta = 0$ for trained models. In Supplementary Fig. 4d,

we plot the results for the Gaussian and sinusoidal regression tasks. We find that the curves visibly degrade when the optical weights are turned off. The curves obtained for $\beta = 0$ are still somewhat nonlinear due to nonlinearities in the non-idealities captured by the *AOC-DT* in the hardware such as signal saturation discussed in Appendix D.

D Non-idealities and their impact on ML performance

In our digital twin, we simulate several signal-chain non-idealities that cause a deviation from equation 2. Some of the non-idealities are condensed into a single mathematical operation inside the *AOC-DT*. For example, we measure the per-channel response of the TIAs and the photodiodes and combine this effect into a per-channel coefficient that is applied before the matrix multiplication (MM) in equation 2. Due to the position of this coefficient within the equation, we refer to this effect as the MM-input coefficient. Similarly, we measure the effective weight that the SLM imparts onto the signal as a function of the raw weight that we sent to the SLM. Weights in SLMs, which are passive optical elements, are bounded by one as mentioned in the Methods. We calibrated and linearized the SLM response (see corresponding discussion in Methods and Supplementary Information, and Supplementary Fig. 2). However, some amount of nonlinearity here remains. In addition, even at the darkest state, the SLM still transmits a fraction of the incoming microLED light, leading to what term the "SLM darkness" offset. Together with the nonlinear SLM response, we condense this effect into the weight distortion non-ideality modelled in the *AOC-DT* .

Overall, the modelled non-idealities include (1) an analog approximation to the $\tanh$ function, (2) a nonlinearity in the microLED response, (3) MM-input coefficients, (4) MM-output coefficients, (5) a combined weight-distortion effect as well as (6) crosstalk across the PBS, and (7) a saturation for larger signal values.

Using our digital twin (DT), we are able to ask "what if" questions and study the impact of certain non-idealities, for instance weight distortion, or the PBS crosstalk. In Supplementary Fig. 5a, we study AOC-ML performance in which one or several non-idealities are turned off in the *AOC-DT* during training. The trained models are then subsequently tested on *AOC*. We choose the Gaussian regression task as benchmark due to the sensitivity of regression tasks. We find that taking into account the input and output scale of the analog $\tanh$ is crucial for the performance of the model. The weight-distortion effect is the second-most impactful non-ideality. Interestingly, we find that the input efficiencies, output efficiencies, and PBS crosstalk each can be turned off individually without significant performance deterioration. However, turning all non-idealities off except the $\tanh$ degrades performance more than just turning off weight distortion. This suggest that non-idealities are not additive in their impact on the model performance and can interact with each other. In Supplementary Fig. 5b, we plot the curves obtained for each ablation study.

D.1 Signal-to-noise ratio (SNR)

The *AOC-DT* can model analog noise by adding signal-dependent Gaussian noise with a given SNR. However, we do not list this as a non-ideality above since noise is inherent to analog computation. In panel Supplementary Fig. 5c, we plot the signal-to-noise ratio (SNR) measured on the *AOC* hardware as a function of signal strength and β-gain. We chose channel 3 as an example to conduct the study on.

**Model**	**Dataset**	β
AOC-256	MNIST	9.02
AOC-4096	MNIST	9.48
AOC-256	FashionMNIST	25.53
AOC-4096	FashionMNIST	10.58
AOC-256	Gaussian Regression	14.62
AOC-256	Sinusoidal Regression	19.89

Supplementary Table 6: β-values obtained for the various *AOC* equilibrium models.

We find the SNR overall to be located in the range of $10 - 15$ dB, but it can drop lower for low signal amplitudes and high β-gains. The β values for our various models are given in 6. As can be seen from 6, we operate towards the higher end of β in Supplementary Fig. 5c. Typical values for the signal are in the range of -2 to 2 V (see e.g., Extended Data Fig. 5a,b,c).

Whenever we train ML models, we do not add noise to the signal digitally in the *AOC-DT* as it prolongs the training process since detecting convergence becomes difficult (see Appendix F). Instead we rely on the natural robustness of equilibrium models discussed in Appendix F.

D.2 Non-ideality Details

In Supplementary Fig. 5d, we plot a fit of the analog $\tanh$ against a rescaled standard $\tanh$ function. The weight distortion effect is modelled with two second-order

polynomials per channel (i.e., 16 separate polynomials for each signal arm) that map the raw neural weight to the effective weight that is used in the VMM operation, see Supplementary Fig. 5e. The PBS we use to split the positive and negative signal paths is not perfect and some light may leak from one signal arm to the other. We model this effect using 2×16 second-order polynomial functions (16 for each signal arm) that map the input signal to the output signal plotted in Supplementary Fig. 5f. The microLEDs have a nonlinear response to the driving current, which we to take into account and model as a third-order polynomial per signal arm. In Supplementary Fig. 5g, we plot a the fit of the microLED response, showing a largely linear response over the current range of interest. In Supplementary Fig. 5h, we plot the aforementioned measured per-channel input efficiencies for the positive and negative signal arm. The output efficiencies are scalar values per signal arm $E^{+}_{\text{output}} = 0.65$ and $E^{-}_{\text{output}} = 1$. The signal saturation non-ideality that occurs in the analog domain in addition to the $\tanh$ nonlinearity is modelled as a piecewise sinusoidal function. We plot the saturation function in Supplementary Fig. 5i. While this saturation should, in principle, be applied at every iteration, we are unable to measure the exact saturation occurring with the loop. Instead, we apply the above saturation at the end after convergence as an effective cumulative saturation. We found that fidelity to the *AOC-DT* can be further improved by limiting the DEQ input to the range -1.3V to 1.3V. This can be achieved either through clipping the values or regularizing the model with a hinge loss to stay within this range. We found that both methods yield similar results but used the clipping method for the results presented in the main text.

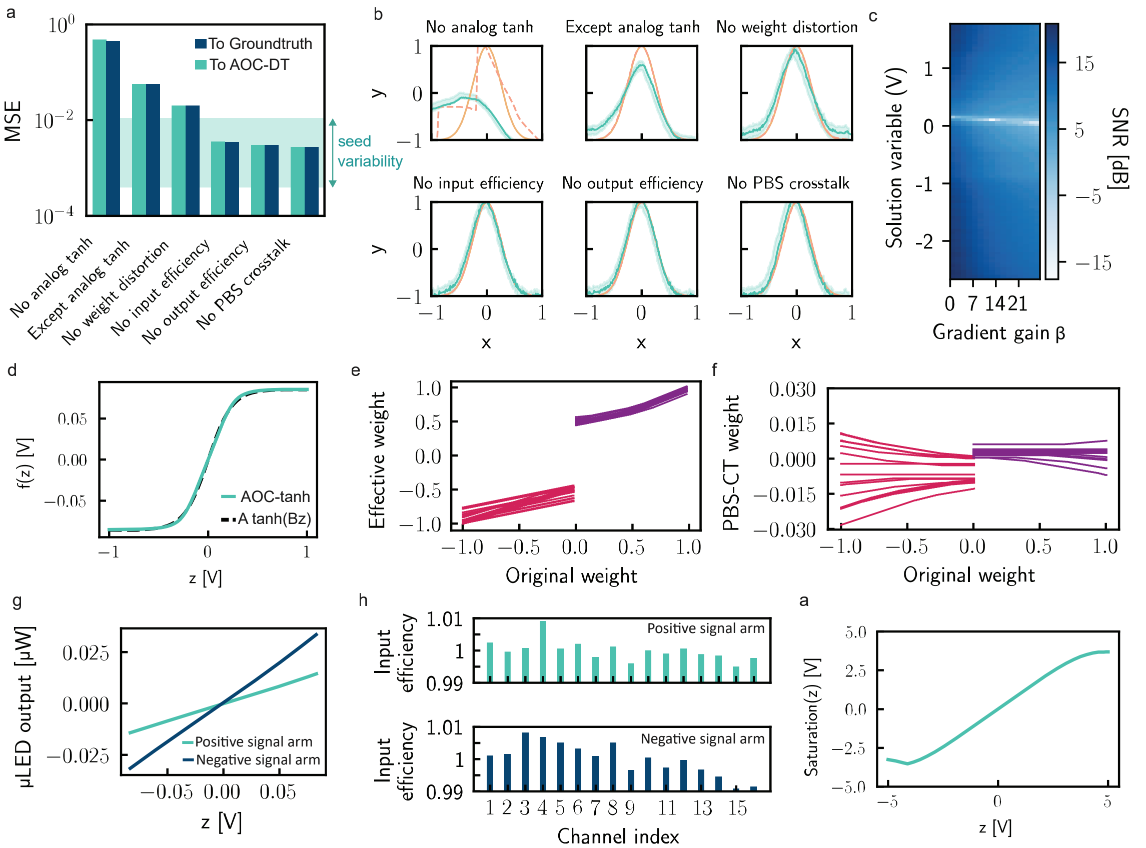

Supplementary Fig. 5: Non-idealities and their impact on equilibrium model Gaussian regression. a, Ablation study of non-idealities deactivated during training in the *AOC-DT* and inference on *AOC*. b, Regression curves obtained for each ablation study. c, Measurement of SNR in dB as a function of signal strength (y-axis) and β-gain (x-axis). We chose channel 3 here as an example to conduct the study on. d, Fit of the analog $\tanh$ against a rescaled standard $\tanh$ function. e, Distorted weights as a function of ideal weights on [0, 1] for both signal arms. f, Weight-distortion used to calculate impact of PBS-crosstalk per channel. g, Fit of the microLED response as a function of driving current. h, Measured per-channel input efficiencies. i, Saturation function used in the *AOC-DT*.

In order to test the robustness of the *AOC-DT* we trained 100 independently initialized models on the Gaussian regression task and tested them on the AOC

device. In Extended Data Fig. 5e, we plot the mean-squared error (MSE) of the *AOC-DT* and *AOC* device towards the ground truth Gaussian curve as a function of each other. We find that the error of the *AOC-DT* towards the ground truth is predictive of the error the device makes against the ground truth and vice versa. The errors fall largely onto the identity line.

E Repeated runs and averaging

The AOC device is inherently noisy due to the analog nature of the computations. We studied how repeating *AOC* runs with the same input and averaging the outputs increases the precision of outputs. Repeated runs linearly increase the cost of computation, creating a trade-off between accuracy and efficiency. We study this trade-off in the curve regression task where it is most visible. In Extended Data Fig. 5a, we plot the MSE between the *AOC-DT* and ground truth Gaussian curve. Both MSEs decrease with the number of runs but converge. The MSE between the ground truth edges even slightly lower, however, we attribute this to chance since parts of the curve can approach the ground truth slightly closer than the digital twin curve. We can average out noise by repeating the full equilibration procedure. Once the noise is averaged out sufficiently, the MSE towards the *AOC-DT* curve as well as the ground truth start to stagnate. Performance beyond this point is either limited by the DT-to-reality gap or the DTs capacity to learn the ground truth curve.

While the MSEs stagnate early, the smoothness of the produced curve Extended Data Fig. 5b continues to improve with the number of runs. This is due to a simple mathematical effect: one can obtain good MSEs with fast-changing curves centered around the ground truth curve that are not smooth. As we find here, however, increased sampling of the curve will lead to a smoother curve. In Supplementary Fig. 6c, we plot example traces of the Gaussian regression for 1, 3, 8, and 11 repeats.

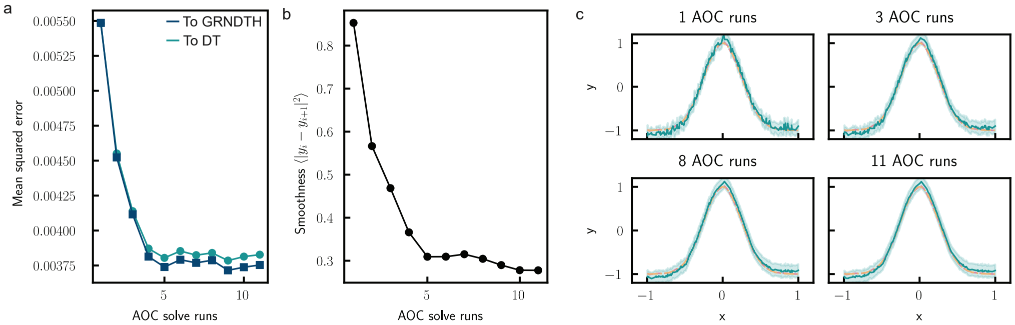

Supplementary Fig. 6: Repeatedly solving the same input and averaging the outputs can lead to reduced variance. a, Mean-squared errors against *AOC-DT* and ground truth as a function of *AOC* runs that are averaged over. b, Smoothness (distance to next point on the curve) as a function of *AOC* runs. c, Example traces of the Gaussian regression for 1, 3, 8, and 11 repeats.

F Increased Out-of-Distribution Generalization and Equilibrium Model Robustness

Equilibrium models have been shown to exhibit increased out-of-distribution (OOD) generalization on various tasks.[11,12] The intuition behind this behavior is that unlike

standard fixed-layer feedforward models, equilibrium models are able to 'think' recursively and are therefore able to learn algorithms rather than shortcuts.[13] In Extended Data Fig. 6a-c, we demonstrate that with the limited instruction set on *AOC*, we would be able to replicate strong OOD generalization from.[11,12] The models trained here are larger and therefore do not fit onto the machine but all follow the same architecture as the DEQ model presented in the main paper: A DEQ model with only a $\tanh$ nonlinearity sandwiched between linear input and output projections. However, as discussed in the main text and Fig. 3, the models considered here have multi-layer unit-cells which, in principle, can be achieved on *AOC* (see Fig. 3). Furthermore, for the prefix-sum task and maze-solving task, we used 1D- and 2D-convolutional models respectively. Again, convolutions could, in principle, be run on AOC by lifting the convolutions into a structured Toeplitz-matrix.

F.1 Addition Task

The model's generalization ability in numerical arithmetic, as depicted in Extended Data Fig. 6a, is evaluated using an addition dataset referenced in.[12] This dataset consists of $60\,000$ samples, each featuring a pair of numbers drawn from a uniform distribution within a defined scale. While training samples are drawn from $\mathcal{U}[-0.1, 0.1]$, test samples include a broader range of scales from 0.1 to 500. The DEQ architecture implemented for this task is based on the feedforward architecture described in[12] and has an input dimensionality of 800 to accommodate two 20×20

matrices representing the addends. It includes three hidden layers, each with 512 neurons, followed by an output layer of dimensionality of 400. The model uses exclusively $\tanh$ activations. As a baseline we employ the feedforward architecture from,[12] but with $\tanh$ nonlinearity for the intermediate layers.

F.2 PrefixSum Task

The model's generalization ability to sequence datasets, as depicted in Extended Data Fig. 6b, is evaluated using a parity-check dataset referenced in.[11] This dataset is composed of binary sequences, where the goal for the network is to determine the cumulative parity, $p_t = \left(\sum_{t'=1}^{t} x_{t'}\right) \mod 2$ with $x_t \in \{0, 1\}$ for each time step t. During training, the model is exposed to binary strings of length 32. The generalization capabilities are then assessed across a range of binary string lengths, specifically 16, 18, 20, 24, 32, 64, 72, 128, 256, and 512 to examine the model's performance on sequences both shorter and longer than those seen during training. The DEQ model architectures for both the DEQ and feedforward models consist of a one-dimensional convolutional layer (conv1d) with 6 hidden channels and a kernel size of 3, followed by a $\tanh$ activation function.

F.3 Maze solving task

The model's generalization ability for image datasets, as depicted in Extended Data Fig. 6c, is evaluated using a maze dataset referenced in,[11] which is designed to identify the correct pathway through a 2D maze. The models are trained on a set of 9×9 mazes to learn the classification of paths. Subsequently, the model's generalization is put to the test with a diverse array of maze sizes in the test set, including dimensions from 9×9 up to 59×59, thereby covering a broad spectrum of complexities. In the DEQ model, a residual block architecture is employed, as described in,[11] featuring two convolutional layers, each with 128 hidden channels, and a $\tanh$ activation function. For the feedforward model, we mirror the DEQ architecture and use a $\tanh$ nonlinearity.

F.4 Noise Robustness

In Extended Data Fig. 6b, we study the robustness of DEQ and feedforward models trained without noise when exposed to various levels of noise (SNR in dB). We note that equilibrium models usually take 10–100 iterations to converge and thus incur a higher total noise amount than their feedforward counterparts. In the figure, we vary the number of layers in the feedforward model but keep the recurring cell of the DEQ to a single layer for simplicity of comparison. For each layer number in the feedforward model, we match the number of parameters in the DEQ by resizing the

single layer appropriately.

We find that for shallow feedforward models, the feedforward architecture performs slightly better at low-noise levels. However, the DEQ model starts to outperform when model sizes increase. Interestingly, feedforward models with more than 3 layers seem to exhibit a local maximum of sensitivity around the 10 dB mark.

This study does not aim to generally conclude that DEQs are always more noise robust then feedforward models. The increased number of iterations of DEQs compared to feedforward models typically also means they incur a larger total amount of noise on analog devices. In addition, at high noise levels it is difficult to ascertain when the DEQ model actually reaches convergence. However, this study shows that DEQs can be more noise robust than feedforward models in certain regimes despite them incurring larger amounts of noise. We capped the total number of iterations for DEQ models at 100 iterations.

G *AOC* for optimization

G.1 Algorithmic approach for optimization

Optimization techniques may be classified into derivate-free methods and algorithms exploiting information about the gradient of the objective function, i.e., gradient-based methods. The *AOC* algorithmic approach can be viewed as the advanced gradient descent method with annealing and momentum. The intuition behind the momentum-based methods is simple: if one assumes that the system state vector s_t represents coordinates of particles, then the momentum parameter is equivalent to the mass of particles moving through a viscous medium in a conservative force field.[14] The annealing schedule makes the system non-conservative: it characterizes the system dissipation rate and controls how much the amplitude of the state s_t is reduced at each time iteration t. The *AOC* algorithm is of a general kind and can be applied to any objective function $F(\mathbf{s})$, although in this study we consider it for solving *QUMO* and *QUBO* problems. The distinction of the *AOC* algorithm is the simultaneous inclusion of both momentum and annealing terms, which dramatically improve the performance of the standard steepest gradient descent method on nonconvex optimization problems. As shown schematically in Supplementary Fig. 7a, the annealing schedule $\alpha(t)$ enhances exploration over multi-dimensional objective function space: it suppresses the contribution from the gradient of the objective function during initial time iterations, thereby flattening the objective

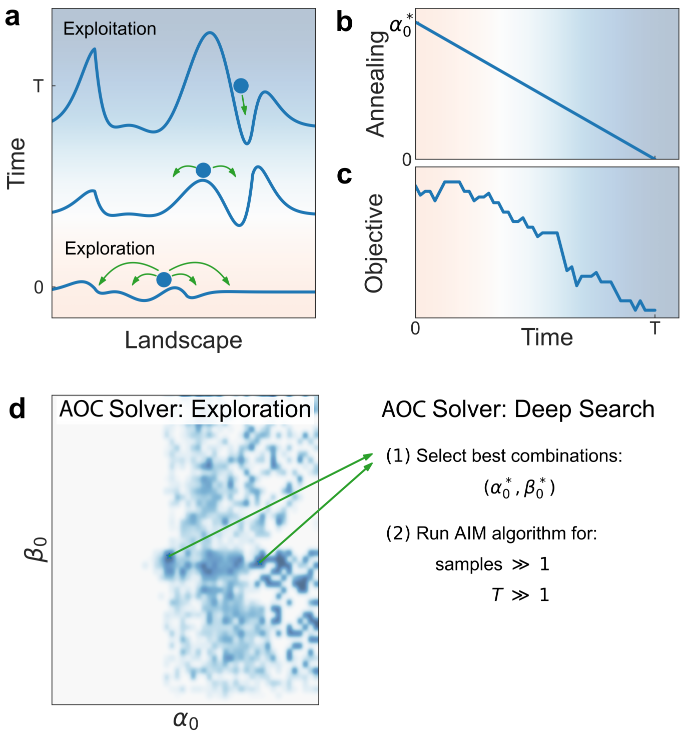

Supplementary Fig. 7: Analog Optical Computer (*AOC*) algorithmic approach. The operational principles of the *AOC* algorithm are depicted schematically for a single combination of parameters in a-c. a, The evolution of the objective landscape is shown for time iterations $t \in [0, T]$. The initially flattened landscape facilitates exploration of the multidimensional variable space and eventually returns to its original form, when exploitation occurs and the algorithm converges to the minimum of the objective. b, The annealing term is characterized by $\alpha(t)$ that decreases linearly over time, ensuring exploration and exploitation stages of the algorithm. c, The better objective values are generally obtained towards the final time iteration T as the contribution of the objective term β increases relatively to the annealing term $\alpha(t)$. d, The two phases of the *AOC* approach are illustrated. During the 'exploration phase', the *AOC* algorithm is simulated for a large number of parameter combinations (α_0, β_0) with small number of time iterations and samples per each combination. During the 'deep search' phase, the parameter pairs (α_0^*, β_0^*), which produce relatively better objective values during exploration phase, are selected and the *AOC* algorithm is simulated for a large number of time iterations and samples per each pair of parameters.

profile. According to the above physical interpretation of the momentum term, the massive particles accelerate their motion in long and narrow valleys, improving the convergence of the iterative approach to minima and providing mechanism for escaping from local minima. From a numerical perspective, the presence of the momentum term increases the range of time step values for which the system converges.[14] In ML, momentum-based approaches are known to greatly improve the speed of training, while the annealing schedule is reminiscent of slowly decaying weights and could be seen as the regularization technique.

Besides the heavy-ball method, one may modify the *AOC* algorithm to be based on, for example, the Nesterov momentum method, in which case the update rule would be given by the following equation:

$$\mathbf{s}_{t+1} = \mathbf{s}_t + \Delta t \left[-\beta \nabla F(\mathbf{s}_t + \gamma(\mathbf{s}_t - \mathbf{s}_{t-1})) - \hat{\alpha}(t)\mathbf{s}_t + \gamma(\mathbf{s}_t - \mathbf{s}_{t-1}) \right]. \quad (5)$$

Unlike heavy-ball method, the Nesterov momentum update evaluates the gradient at a point $(\mathbf{s}_t + \gamma(\mathbf{s}_t - \mathbf{s}_{t-1}))$, to which the momentum term has been applied, instead of evaluating the gradient at the most recent state $\mathbf{s}_t$. The performance comparison between *AOC* algorithms based on different momentum-based methods is a promising avenue for future studies.

The general tendency of the *AOC* algorithm to achieve better objective values towards the final time iteration T is ensured by relatively increasing contributions from the gradient of the objective with respect to the annealing and momentum

terms. The annealing term may be time-dependent in either a linear or nonlinear way although here we consider the linear schedule $\hat{\alpha}(t) = \alpha_0(1 - t/T)$, as shown in Supplementary Fig. 7b. Hence, the annealing term decreases to zero over time and the momentum term vanishes for the equilibrium solution, which means that the *AOC* algorithm finds a solution corresponding to the minimum of the objective function $F(\mathbf{x})$ at time iteration T. As mentioned in the main part of the paper, having such explicit stopping criteria is a lucid advantage of the *AOC* algorithm for an all-analog hardware implementation, as it avoids the complexity of multiple intermediate readouts that stochastic heuristic approaches suffer from.[15]

The impact of alternative nonlinearities on computational performance has been investigated for optimization. While different nonlinear functions can influence solver efficiency, we found that a small set, including $\tanh()$, $\sin()$, and $\cos()$, perform similarly. More importantly, the addition of annealing and momentum terms led to substantial algorithmic improvements, significantly enhancing optimization performance.

G.2 *AOC* hardware performance in optimization

We conduct a comprehensive evaluation of the *AOC* hardware on a diverse set of *QUMO* and *QUBO* synthetic instances in the main part of the manuscript. To get these results, we vary the parameters (α_0, β_0) within their accessible range in hardware. Given the novel nature of the *QUMO* abstraction, there is a lack of research on

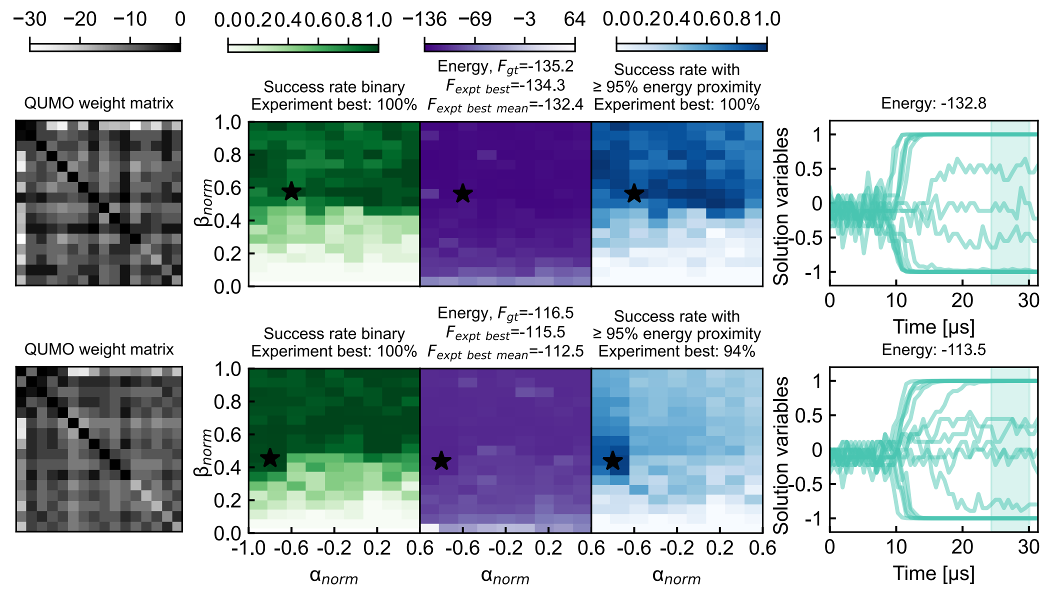

Supplementary Fig. 8: Analog Optical Computer (*AOC*) for Optimization: *QUMO*. Two rows show the performance of the *AOC* hardware on two *QUMO* instances with 16 variables. The first column depicts the weight matrix structure of the *QUMO* instances. The second column shows the success rate map over binary variables only, the energy map, and the success rate map to achieve energy over 95% of the best known solution. All maps are shown for the varying normalized hardware parameters $(\alpha_{norm}, \beta_{norm})$, which are linear transformations of algorithmic parameters (α_0, β_0). The third column shows the variable time traces for the optimal parameter combination indicated by star symbols in the maps. The *QUMO* instances contain 4 and 5 continuous variables.

methods for generating challenging small size instances with a mixture of binary and continuous variables. We develop a technique for planting random continuous minimizer values in the global solution and generate 50 *QUMO* instances with 16 variables, which due to quantization process and random perturbations may become more challenging to solve. As an example, the detailed results are shown for two *QUMO* instances in Supplementary Fig. 8. For each instance, we present the weight

matrix structure, the success rate map over binary variables only, the objective (or energy) value map, the success rate map to achieve energy over 95% of the best known solution, and the variable time traces for the optimal parameter combination.

Given the historical emphasis on the *QUBO* model, we consider 50 *QUBO* instances with 16 variables, representing problem classes that are known to be computationally challenging at scale. These instances are divided equally between two graph topologies, namely dense fully-connected and sparse three-regular graphs. In both cases, the matrix weight elements are drawn from the Gaussian distribution and their bit precision is reduced to 8 bits or lower, resulting in instances belonging to the Sherrington-Kirkpatrick[16] and weighted maximum cut problems. Similar to the detailed results for the two *QUMO* instances above, the results for the two *QUBO* instances are shown in Supplementary Fig. 9.

We note that the success rate, a widely used metric for evaluating the performance of heuristic solvers, is defined as the probability of finding the global objective value for a given set of parameters. The hardware solver targets exact objective values for the *QUBO* instances while the relative 95% proximity is set for *QUMO* instances to account for hardware imperfections. In both cases, the results are obtained by averaging variable states over the shaded regions in time traces and computed in an entirely analog manner without digital pre- and post-processing. As demonstrated in the main part of the manuscript, we can randomly sample various parameter combinations (α_0, β_0), taking samples from them, and therefore estimate what is the total number of samples required before the *AOC* hardware can achieve

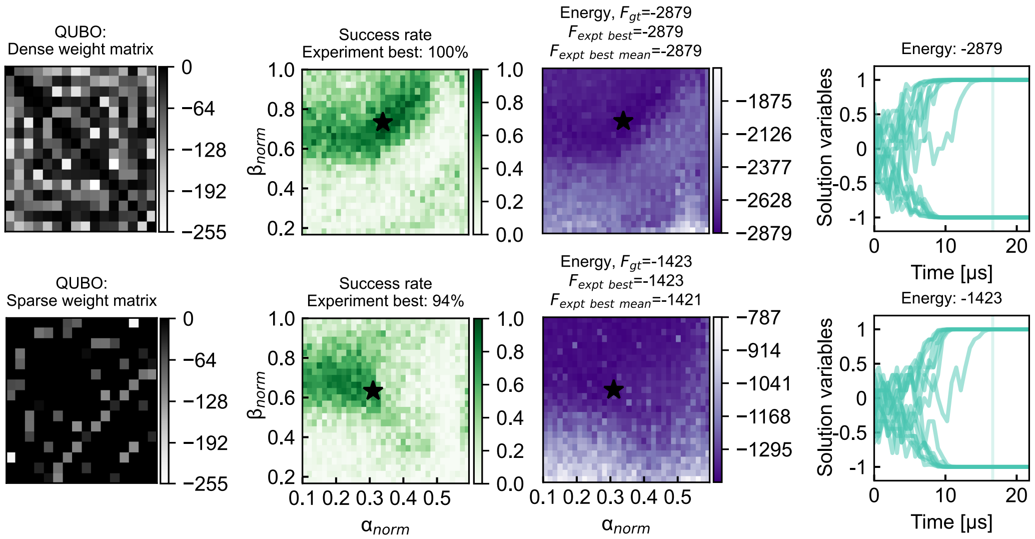

Supplementary Fig. 9: Analog Optical Computer (*AOC*) for Optimization: *QUBO*. Two rows show the performance of the *AOC* hardware on two *QUBO* instances with 16 variables. The first column depicts the weight matrix structure of the *QUBO* instances, dense and sparse (3-regular). The second column shows the success rate map over binary variables., The third column shows the energy map. Both maps are shown for the varying normalized hardware parameters $(\alpha_{norm}, \beta_{norm})$, which are linear transformations of algorithmic parameters (α_0, β_0). The forth column shows the variable time traces for the optimal parameter combination indicated by star symbols in the maps.

Success Rate [%]

	Instance 1	Instance 2	Instance 3
AOC	100%	100%	100%
Quantum	42%	58%	43%

Supplementary Table 8: Analog Optical Computer (*AOC*) for Optimization: Transaction Settlement. The table shows the *AOC* success rates for three *TS* scenarios formulated as *QUMO* instances, with quantum hardware results reproduced from.[18]

100% proximity for the *QUBO* instances and 95% proximity for the *QUMO* instances. We note that the consideration of the relative proximity to the best known solution within several percent is a common practice when evaluating the performance of non-traditional hardware platforms or heuristic optimization methods,[17] as the exact solutions are often inaccessible due to the system noise in the former and due to much harder to solver large-scale instances for the latter.

We present the success rates for small size transaction settlement (*TS*) scenarios, derived from real settlement data,[18] in 8. We note that the quantum hardware is capable of solving only *QUBO* instances. Consequently, the solution process for these *TS* scenarios involved iteratively determining binary variables using quantum hardware while holding continuous variables fixed, updating the continuous variables in the digital domain while keeping the binary variables fixed, and repeating this process until convergence. In contrast, *AOC* addresses these *TS* instances, reduced to *QUMO* instances with eight variables after pre-processing, in a single run.

G.3 Medical image reconstruction

For the reconstruction of the Shepp-Logan phantom slice, we consider a realistic scenario where the system operator $\mathbf{A}$ is represented as $\mathbf{A} = \mathbf{MFW^T}$, where $\mathbf{M}$ is the undersampling mask, $\mathbf{F}$ is the discrete Fourier transform matrix, and $\mathbf{W}$ is the wavelet transform matrix. We note that the reconstruction problem is formulated over real variables in the main part of the paper, which means that the imaginary part of the complex-valued operator $\mathbf{A}^\dagger\mathbf{A}$ is not considered. In practice, one can also generalize the reconstruction problem to complex variables, which would require optimizing the following objective:

$$\min_{\mathbf{z}} \frac{1}{2} \|\mathbf{y} - \mathbf{Az}\|_2^2 + \lambda_1 \mathbf{1}^T \boldsymbol{\sigma} + \lambda_2 (\mathbf{1} - \boldsymbol{\sigma})^T |\mathbf{z}|^2. \tag{6}$$

where $|.|^2$ is element-wise. We show additional details for the reconstruction of the Shepp-Logan phantom slice, formulated as a 64-variable *QUMO* instance, in Supplementary Fig. 10. Over the block coordinate descent steps, the objective value decreases for the *AOC* hardware as shown in Supplementary Fig. 10a. The reconstructions of the Shepp-Logan phantom line in pixel space are shown for the *AOC* hardware as well as for the minimization of the data fidelity term only in Supplementary Fig. 10b, with both compared to the reference via mean-squared error (MSE). The binary variables of the *QUMO* instance are shown for the *AOC* hardware in Supplementary Fig. 10c. The reconstructions in the wavelet domain are shown for the *AOC* hardware as well as for the minimization of the data fidelity

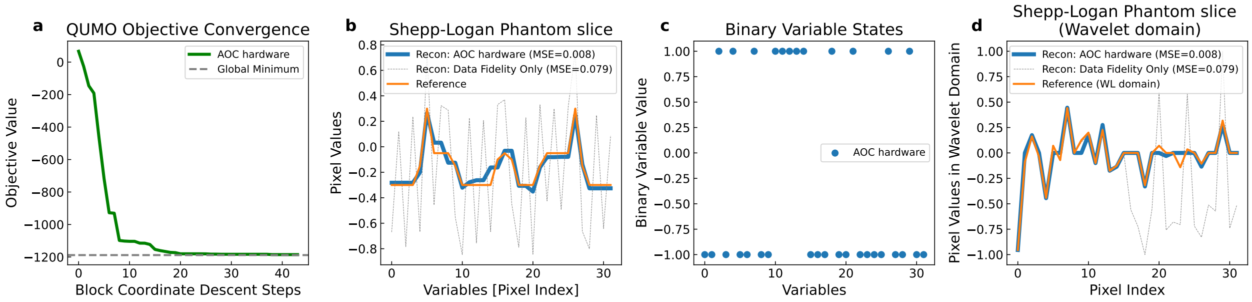

Supplementary Fig. 10: Analog Optical Computer (*AOC*) for Shepp-Logan Reconstruction. a, The objective value decrease over block coordinate descent steps is shown for the reconstruction of the Shepp-Logan phantom slice, formulated as *QUMO* instance and solved by *AOC* hardware. b, The reconstructions of the Shepp-Logan phantom slice in the pixel space are shown for the *AOC* hardware and minimization of the data fidelity term with both compared to the reference via mean-squared error (MSE). c, The binary variables of the *QUMO* instance are shown for the *AOC* hardware. d, The reconstructions in the wavelet domain are shown for the *AOC* hardware and minimization of the data fidelity term with both compared to the reference via mean-squared error (MSE). The continuous variables of the *QUMO* instance correspond to the pixel values in the wavelet domain.

term only in Supplementary Fig. 10d, with both compared to the reference via mean-squared error (MSE). We note that the actual continuous variables of the *QUMO* instance correspond to the pixel values in the wavelet domain.

We also show the relative proximity to the best known solution for optimizing individual *QUMO* instances over block coordinate descent steps in Supplementary Fig. 11. For each BCD step or, equivalently, for each *QUMO* instance, the relative proximity is shown for the *AOC* hardware over the different threshold sets for the success rates.

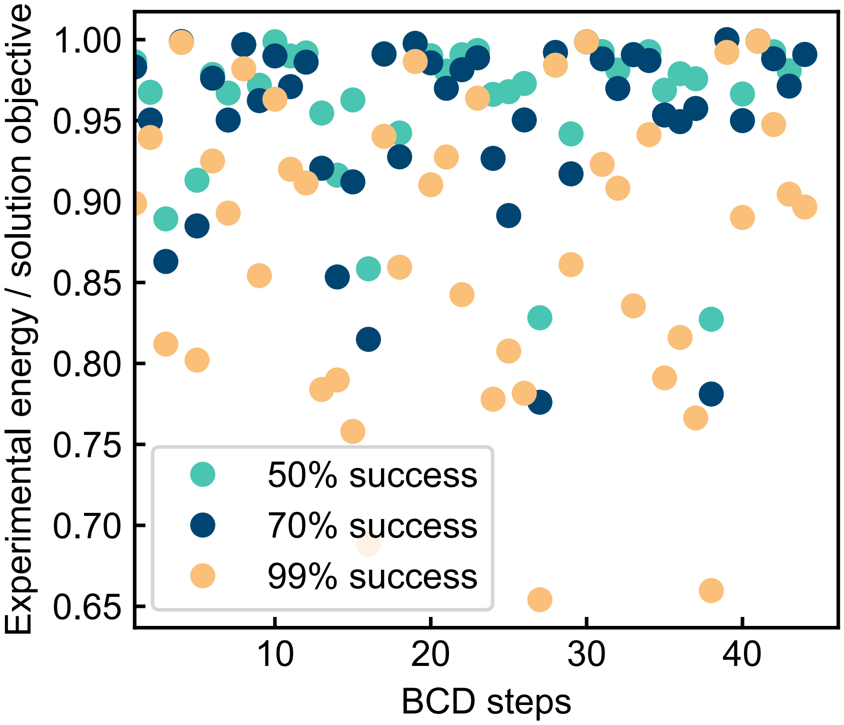

Supplementary Fig. 11: Analog Optical Computer (*AOC*) for Shepp-Logan Reconstruction: Relative *QUMO* proximity. The relative proximity to the best known solution for optimizing individual *QUMO* instances, which correspond to the block coordinate descent steps, is shown for the *AOC* hardware over the different thresholds set for the success rates. As expected, for the lower thresholds of the success rates, the relative proximity is higher.

G.4 *AOC* parameters for optimization

Optimizing heuristic methods for solving optimization problems often requires careful calibration of multiple hyperparameters. While external optimization tools or grid search are commonly used, the exponential growth of the hyperparameter space makes calibration time a critical yet often overlooked factor. For the *AOC* algorithm, three key parameters $\{\alpha_0, \beta, \gamma\}$ need tuning. Our simulations show that α_0 and β significantly influence solution quality, while the momentum parameter γ has less impact. A linear stability analysis reveals that scaling β as $\beta = \beta_0 / \lambda_{\max}$ where $\lambda_{\max}$ is the largest eigenvalue of the weight matrix W, keeps β_0 and α_0 in a consistent optimal range across diverse problems.

To streamline the search for optimal parameter values, we propose a two-phase *AOC* solver framework resembling a black-box solver. In the 'exploration' phase, a broad parameter space is sampled to identify promising candidates (α_0, β_0). These are refined in a 'deep search' phase as illustrated in Supplementary Fig. 7d.

G.5 *AOC-DT* performance at scale in optimization

Here we validate the algorithmic performance of the *AOC* solver that is implemented on the GPU. We consider a comprehensive set of benchmarks, one of which is the quadratic programming library (QPLIB). The QPLIB benchmark is a collection of

challenging synthetic and real-world problems, gathered over a year-long open call from mathematical and numerical analysis communities.[19,20] For evaluating the *AOC* solver on *QUMO* problems, we focus on a subset of QPLIB benchmark, namely the non-convex problems with linear inequality constraints. These problems have up to several thousand variables, while the number of constraints reaches ten thousand. These constraints lead up to ten thousand additional continuous variables for problems formulated within the *QUMO* abstraction. We consider unconstrained and equality-constrained binary problems within QPLIB. In addition, we use the well-studied G-Set benchmark with synthetically generated problems up to $20\,000$ variables,[21] Tile3D and Wishart instances from a recently introduced CHOOK generator,[22] as well as a set of manufacturing problems, i.e., RCDP.

For *QUMO* problems, we compare the *AOC* approach against the commercial Gurobi solver, that outperforms such optimization packages as Octeract, Baron, and Scip.[23] For *QUBO* benchmarks, in addition to Gurobi, we consider two heuristic approaches, namely simulated annealing and parallel tempering, that are known for a consistently better or similar performance over other physics-inspired methods.[24–26] Both heuristic approaches benefit from highly-optimised implementations in Azure quantum inspired optimization service and work as black-box solvers for a given time limit.[27]

We design the *AOC* approach to resemble a black-box solver operation with the main sensitive parameters (α_0, β_0) dynamically adjusted for a given time limit for each problem, while small variations of the momentum parameter values are explicitly

mentioned within and between benchmarks. For a representative performance comparison across such a disparate set of benchmarks, we consider the quality of solution improvement metric:

$$\text{Objective Improvement} = \begin{cases} 100\% \cdot \frac{F_{\text{AOC}} - F_{\text{Best rest}}}{F_{\text{Best known}} - F_{\text{Best rest}}}, & \text{if } \textit{AOC} \text{ is better than all competing methods} \\ 0\%, & \text{if } \textit{AOC} \text{ is equal to the best competing method} \\ -100\% \cdot \frac{F_{\text{Best rest}} - F_{\text{AOC}}}{F_{\text{Best known}} - F_{\text{AOC}}}, & \text{if } \textit{AOC} \text{ is worse than the best competing method} \end{cases} \tag{7}$$

where $F_{\text{Best known}}$ represents the best known minimum of the objective function for the problem and the *AOC* algorithm is compared against the best solution found by competing solvers: Gurobi, parallel tempering, or simulated annealing. Since all solvers are given the same computational resources equivalent to about 100 seconds of the *AOC* solver, the solution improvement metric serves as a good indicator of their relative performance in terms of finding better objective function values. We note that relying only on the success rate as the main metric could be misleading due to the often-overlooked effort in tuning hyperparameters. This is why our digital twin solver is designed as a black-box optimizer, incorporating parameter search within the given time budget.

Comment on objective improvement metric. The introduced objective improve-

ment metric can be used evaluate the relative improvement in objective value found by the solver of choice, i.e., the *AOC* algorithm, compared to other competing methods. In particular, the objective improvement of 100% happens when the *AOC* approach finds the best known objective while the competing solvers cannot achieve it, and in the reverse situation, the objective improvement is -100% when one of the competing solvers finds the best known objective while the *AOC* solver could not.

For the *QUMO* benchmark, we report the *AOC* speed-up against the Gurobi solver on the hardest quadratic binary problems with linear inequality constraints within QPLIB (QPLIB:QBL). Since Gurobi attempts not only to find the optimal solution, but also to prove the global optimality of the solution, we consider the Gurobi time when it first finds the best objective value, which can be compared with the time of the *AOC* solver, that provides no global optimization guarantees. In the main part of the paper, we consider ten of the most difficult instances requiring more than a minute of computational time for Gurobi to find the best known solution. The one minute threshold is chosen as the problems that can be solved faster can be seen either intrinsically simple or their structure could be substantially simplified by the pre-processing techniques of Gurobi. The *AOC* solver is up to three orders of magnitude faster in all *QUMO* except the two instances, one of which it is unable to solve. Moreover, the *AOC* solver finds the new best solutions for two heavily constrained instances in about 40 seconds: the instances 3584 and 3860 have about 500 binary and 10000 continuous variables in *QUMO* formulation. To evaluate the speed-up, we run Gurobi for these two instances for five days. For the instance 3584,

Gurobi finds the same solution as the *AOC* solver in about 54000 seconds, while proving its global optimality takes four and half days. The Gurobi solver optimises instance 3860 to the same quality as the *AOC* approach in about 13000 seconds with an optimality gap of 8% in five days.

Although we do not know the origin of the hardest instances discussed above, we notice that the ones solved by the *AOC* approach are united by the same type of inequality constraints, which can be seen as Horn clauses.[28] The Horn clauses play fundamental role in automated theorem proving and, intuitively, imply that if all variables except one are true, then that last variable must also be true. Such insight into the logical properties of this subset of the hardest QPLIB problems may imply that the *AOC* solver can efficiently propagate the logical clauses of Horn type that can bring advantageous performance in solving logic programming problems.

In Supplementary Fig. 12, the *AOC* solver demonstrates consistent objective improvement over the competing methods across several *QUBO* benchmarks including Wishart, RCDP, Tile3D, and QPLIB:QUBO, where QPLIB:QUBO includes a subset of graphs from QPLIB that are natively represented as the *QUBO* model. Remarkably, *AOC* solver finds new best solutions for the two largest problems with over thousand variables from QPLIB:QUBO, namely 3693 and 3850 instances. For Gurobi, the optimization of instance 3850 takes 110000 seconds to reach the same solution quality as *AOC* solver in 40 seconds, with the gap of 3% in 5 days. The instance 3693 cannot be optimised to the similar *AOC* quality of solution by Gurobi in 5 days. The *AOC* solver further demonstrates competitive performance on the G-Set instances. With many

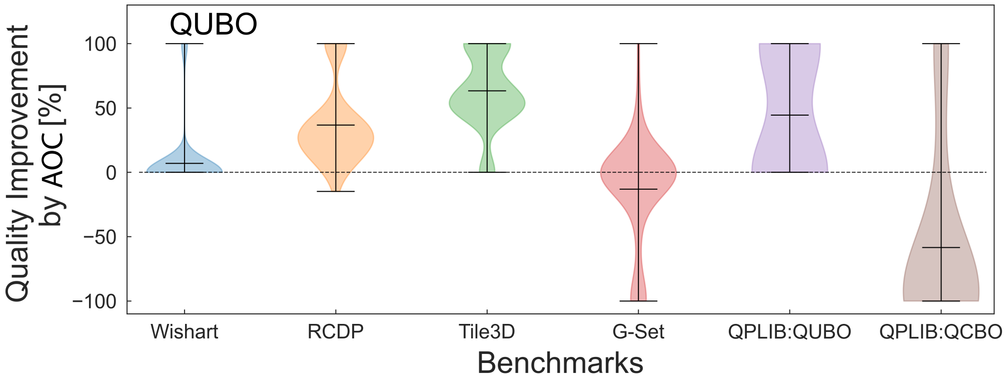

Supplementary Fig. 12: *AOC-DT* Performance: *QUBO*. The violin plots demonstrate distribution of the quality improvement performance for the *AOC* solver compared to the best solution found by competing methods across six *QUBO* benchmarks. The competing methods include parallel tempering, simulated annealing, and Gurobi solvers.

heuristic approaches applied to the G-Set benchmark over the past two decades, the performance of the Gurobi solver has probably been overlooked due to the general perception that it is inefficient at solving natively formulated *QUBO* problems. In our analysis, we observe that Gurobi solves particularly well instances with certain graph topologies, finding new best global solutions for four graphs with torus geometry from G-Set benchmark, although it still underperforms on other *QUBO* instances.

Among all *QUBO* benchmarks, *AOC* solver is behind only on the quadratic constrained binary optimization problems within QPLIB (QPLIB:QCBO), which includes instances with binary variables and equality constraints. We note that Gurobi employs various pre-processing techniques that can drastically reduce the number of variables and constraints,[29] thereby greatly simplifying problems before invoking the main optimization methods such as simplex, interior point, and branch-

and-bound.

With the development of similar pre-processing techniques for the *AOC* solver, the *QUBO* and *QUMO* formulations of constrained problems could be simplified and one could expect an improved performance for the *AOC* approach. For example, we implement one of the pre-processing methods for solving two *QUMO* instances within QPLIB:QBL benchmark, as mentioned in the main part of the paper.

G.6 Competing solvers in optimization benchmarks

For a fair comparison, we ensure that all methods use similar computing resources. Although the implementation of GPU or CPU based solvers can require highly varying engineering efforts, we try to estimate the cost of running solvers on the hardware, on which they are designed to run, and vary the time limit across solvers accordingly to ensure similar cost per solver run. In what follows, the *AOC* solver runs on GV100 GPU for $5 - 300$ seconds per instance across all benchmarks. For the highest time limit of *AOC* we estimate time limits of about 400 seconds per instance for the simulated annealing and parallel tempering methods run on multicore CPU machine. In case of Gurobi, our licence allows one to use only up to 8 cores, so it is given 1000 seconds per instance. To simplify the evaluations, the competing solvers are always given these maxed out time limits even for the instances on which *AOC* uses less than 300 seconds.

Simulated annealing. As described in Azure quantum inspired optimization service,[27] simulated annealing is a Monte Carlo search type of method that simulates a state of varying temperatures, where the temperature of a state influences the decision-making probability. For optimization problems, the algorithm starts at an initial high-temperature state where "bad" moves in the system are accepted with a higher probability, and then slowly "cools" on each sweep until the state reaches the lowest specified temperature. At lower temperatures, moves that don't improve the objective value are less likely to be accepted. For *QUBO* problems, each decision variable is "flipped" based on the objective value impact of that flip. Flips that improve the objective value are accepted automatically. Flips that don't improve the objective value are accepted on a probabilistic basis, calculated via the Metropolis Criterion.

Parallel Tempering. As described in Azure quantum inspired optimization service,[27] parallel tempering can be regarded as a variant of the simulated annealing algorithm, or more generally Monte Carlo Markov Chain methods.[30] As with simulated annealing, the cost function is explored through thermal jumps. Unlike simulated annealing, a cooling temperature is not used. Instead of running a single copy of the system, Parallel Tempering creates multiple copies of a system, called replicas, that are randomly initialized and run at different temperatures. Then the same process is followed as in simulated annealing, but based on a specific protocol two replicas can be exchanged between different temperatures. This change can enable walkers that were previously stuck in local optima to be bumped out of them, and thus encourages a wider exploration of the problem space.

Gurobi. Gurobi is a commercial solver that is highly-optimized to work as a black-box solver.[31] Gurobi implements pre-solve techniques that can drastically reduce the input problem size and the number of constraints.[29] We note that Gurobi finds for the first time the global minima solutions to several largest G-Set instances including G62, G72, G77, G81, which are all united by the same torus graph topology, and further proves that the best known solutions are exact for other graphs with torus topology: G11, G12, G13, G32, G33, G34, G48, G49, G50, G57, G65, G66, G67.

G.7 Optimization benchmarks description

QPLIB benchmark. The quadratic programming library (QPLIB) is a library of quadratic programming instances[19] collected over almost a year long open call from various communities, with the selected instances being challenging for state-of-the-art solvers. As described in the main part of the paper, we consider only the hardest instances within the QPLIB:QBL class of problems, which contains instances with quadratic objective and linear inequality constraints, the QPLIB:QCBO class of problems which contains instances with quadratic objective and linear equality constraints, and the QPLIB:QBN class of problems which contains *QUBO* instances.

Wishart benchmark. Wishart planted ensemble (WPE) problems[32] are originally planted binary Integer Linear Programming (ILP) problems whose coefficients are drawn from a correlated multivariate Gaussian distribution. It has been shown that

in the hard regime, the ground state is extremely difficult to find using Monte-Carlo-based algorithms (such as parallel tempering) even for small problem sizes of 32 variables. The statistics have been collected across 100 instances.

RCDP benchmark. Rotationally constrained discrepancy problem (RCDP) arises in the automotive manufacturing industry when one needs to arrange n disks on a common axis.[33] Due to imperfections, the disks have uneven surfaces and we wish to decide the alignment of disks to minimize total height. The disks have uneven surfaces due to imperfect machining. The goal is to rotate the disks to appropriate angles with respect to a reference orientation such that when all put through the common axel, the cumulative surface height in each sector is as close as possible to the ideal case when all surfaces are perfectly flat. This problem can be formulated as either mixed integer programming or QUBO. The RCDPs are tunable in hardness by increasing either n and the number of sectors K or the correlation between the sectors. For our study, the *QUBO* problems were generated by external team. The statistics have been collected across 100 instances with 360 variables.

Tile3D benchmark. The 3d tile planted problems[34] are highly tunable short-ranged Ising planted instances based on partitioning the problem graph into edge-disjoint subgraphs. It has been shown that the tile-planted problems can be made orders of magnitude (in terms of time-to-solution) harder than a typical 3D Gaussian spin-glass instance. The statistics have been collected across 100 instances with 512 variables.

G-Set benchmark. The G-Set benchmark includes a collection of synthetically generated instances from 800 to 20000 variables.[35]

Sherrington-Kirkpatrick (SK) and dense MaxCut problems. We further evaluate the performance of *AOC-DT* on similar inputs as those used in,[36] see Supplementary Fig. 13. Observe that these are optimization problems that use only binary variables, hence, they are typical *QUBO* problems. A variety of physical (Ising) solvers and heuristics have been evaluated on such inputs (see Figures 3 and 4 of,[36] and references therein).

G.8 Problem mapping advantage of *QUMO* abstraction over *QUBO* model

Comment on QUMO abstraction. The introduced *QUMO* abstraction can be seen as a subclass of mixed integer nonlinear programming (MINLP) class of problems. The MINLP problems appear in various fields including engineering design problems, particularly in chemical engineering where complex chemical processes can be modelled using quadratic or other nonlinear functions, and integer variables can represent discrete decisions. These optimization problems have a wide range of applications in areas such as chemical process design and control, network design, planning and scheduling, energy systems, and portfolio optimization. To address these problems, a variety of tailored algorithms have been developed that outperform

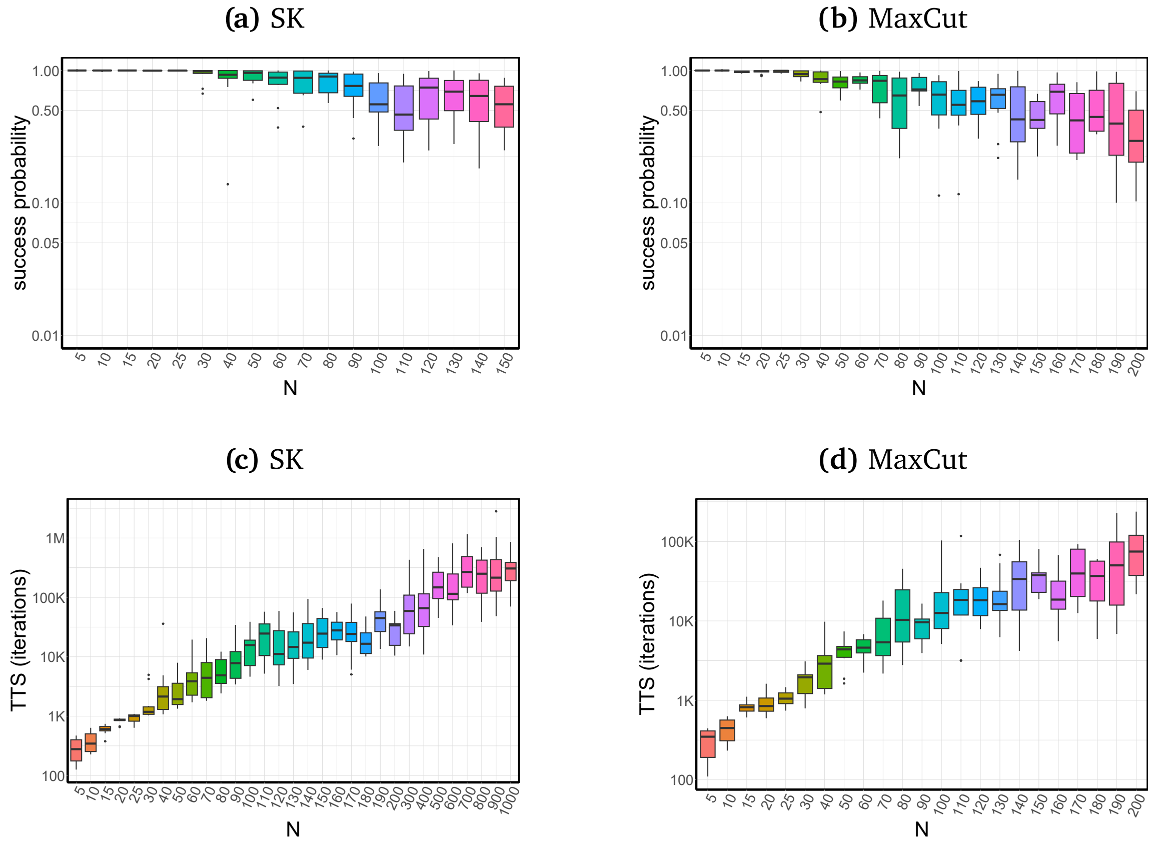

Supplementary Fig. 13: Success probabilities (a and b) and time to obtain a 99% success probability of obtaining the ground state (TTS) (c and d) of the *AOC-DT* for Sherrington-Kirkpatrick (SK) (a and c) and dense MaxCut (b and d) problems, as a function of the problem size (N). Observe the logarithmic scale in the y-axis, and the non-linear x-axis. Graph construction and evaluation follows[36] (Figures 3 and 4). The Ising graphs used as input in a and c are constructed by creating a symmetric $N \times N$ matrix where each non-diagonal entry takes the value -1 or 1 with equal probability (diagonal entries are 0). The MaxCut graphs used as input in b and d are constructed by creating a symmetric $N \times N$ matrix where each non-diagonal entry takes the value 0 or 1 with equal probability; we further reject graphs that are not connected. For each input graph size (N) we construct 10 matrices and provide aggregate performance statistics as box plots. The TTS metrics is reported as the number of required iterations to obtain the ground state with 99% success probability; ground state is defined as the best solution found by the solver. Hence, a lower bound of the actual wall-time is the reported TTS number multiplied by the round-trip time of the system (ignoring all other system overheads).

general-purpose MINLP solvers. Given the increasing number of applications for MINLP and the growing demand for powerful analytics and decision-making tools, significant algorithmic developments are likely to emerge in this area over the next decade, including advances in convexification, decomposition, and parallel implementations to handle large-scale problems arising in machine learning.

Within MINLP, the mixed binary quadratic programming (MBQP) class[37] would include constrained optimization problems with binary and continuous variables, which is often also referred to as mixed binary optimization (MBO) problems.[18] Recent advancements in quantum and quantum-inspired technologies, as well as in optical Ising solvers capable of approximately searching for the ground state of Ising spin Hamiltonians, have increased interest in integrating Ising problems into the process of solving difficult optimization problems. Existing approaches range from direct mapping of MBQP to hybrid quantum-classical methods based on optimization algorithms.[18,37] The problems within MBQP class with linear constraints could be efficiently translated to the *QUMO* abstraction with an additional continuous variable per each inequality constraint, making a wide range of applications more accessible for solving directly on *AOC*.

Advantage of QUMO abstraction over QUBO model. The realistic optimization problems are commonly constrained problems with mixed variable types. The advantage of transforming an optimization problem to *QUMO* abstraction rather than *QUBO* model is evident for a wide class of problems including such with both binary and continuous variables, and problems with inequality constraints. To

illustrate this mapping advantage, we consider a toy quadratic optimization problem with a linear constraint:

$$\text{Original problem :} \quad \min \quad ax_1x_2 + bx_3 \tag{8}$$

$$s.t. \quad 0 \leq cx_1 + dx_3 \leq 1, \tag{9}$$

in which all three variables can be assumed to be binary $x_i \in \{0, 1\}$. To get an unconstrained optimization problem, the inequality constraint can be mapped to the objective by using a penalty method:

$$\text{Unconstrained problem :} \quad \min ax_1x_2 + bx_3 + P_0(cx_1 + dx_3 + s - 1)^2, \tag{10}$$

where $s \in [0, 1]$ is the continuous slack variable and P_0 is a large enough constant that ensures that the constraint is satisfied. A *QUMO* solver can be applied directly to this unconstrained optimization problem. In contrast, if one has a *QUBO* solver, then the additional mapping step needs to take place. To map a continuous variable to binary representation, one may consider either unary or binary encodings:

$$\text{Unary encoding :} \quad s = \sum_{j=1}^{2^{N_{bits}}} y_j \tag{11}$$

$$\text{Binary encoding :} \quad s = \sum_{k=0}^{N_{bits}-1} 2^k y_k, \tag{12}$$

where N_{bits} is the target bit precision for the continuous variable. From this simple analysis, the problem mapping to the *QUMO* abstraction is one-two orders of magni-

tude more efficient than to *QUBO* model in terms of the total number of variables. For the hardware *QUBO* solvers, the available bit precision for the input problem weights needs to be taken into account, which may limit one to use unary encoding. The limitations of *QUBO* for real-world applications have led some optimization platforms to expand their capabilities. Fujitsu's latest digital annealer,[38] for example, allows linear inequality constraints to be specified in its interface. However, the underlying hardware is still a QUBO solver and even at the interface level, continuous variables still require binarization, introducing additional overhead.

G.9 Quantum hardware limitations for optimization problems

Quantum computing has emerged as a candidate hardware solver for hard optimization problems.[39] Similar to the other physical machines, quantum computers also target the *QUBO* abstraction. In addition to sharing the same abstraction limitations, additional quantum hardware constraints further limit the potential of quantum computing as efficient solver for optimization problems. Quantum approximate optimization algorithm (QAOA), that targets quantum gate computers, performs similar to random guess on small-size *QUBO* problems[39] with theoretical estimations that even a million physical qubit hardware will be still many orders of magnitude slower than existing classical heuristics.[40] The quantum computers can offer up to quadratic speed-up over classical alternatives in solving NP-hard optimisation problems, to which *QUBO* and *QUMO* belong. But, this quadratic speed-up further

suffers from the slow time operation of quantum gates. Hence, unless new quantum optimization algorithms emerge and quantum computers scale significantly, it is unlikely that quantum computing will allow us to tackle challenging optimization problems at sizes of interest.

Quantum annealing platforms offer another approach for solving *QUBO* problems. The D-Wave pioneered *QUBO* hardware solvers and managed to scale from tens of variables to the current several thousands of variables over two decades.[41] In practice, one of the main challenges for this hardware is the limited connectivity of only 15 connections per each physical variable, i.e., the Pegasus topology. This translates to additional mapping overhead of *QUBO* problem with an arbitrary topology to the D-Wave machine. In the worst case of the fully-connected graph, the latest D-Wave Advantage hardware with 5000 qubits can accommodate only problem sizes up to 150 variables, significantly limiting scalability and energy efficiency. This one order of magnitude mapping overhead is further amplified by one-two orders of magnitude mapping advantage of *QUMO* over *QUBO* abstraction. D-Wave's Leap hybrid solver mitigates the limitations of the underlying quantum annealing hardware by coupling it with a digital solver that breaks large problems into sub-problems solved separately on hardware before recombining results digitally. However, for problems with dense connectivity, linear inequality constraints or continuous variables, the *QUBO*-limited nature of the hardware still impose significant scaling overheads.

G.10 Physical analogy of the *AOC* algorithmic approach in optimization

AOC algorithm belongs to the family of gradient descent methods that use the concept of momentum. The first momentum method has been introduced in 1964 by Boris Polyak[42] and is known as heavy-ball method or simply momentum method. For optimising the nonlinear function $F(x)$, it could be written as:

$$\mathbf{x}_{t+\Delta t} = \mathbf{x}_t + \Delta t \left[-\beta \nabla F(\mathbf{x}_t) + \gamma(\mathbf{x}_t - \mathbf{x}_{t-\Delta t}) \right], \tag{13}$$

As discussed in the main text of the paper, the *AOC* algorithm is the advanced gradient descent method represented by the iterative update rule:

$$\mathbf{x}_{t+\Delta t} = \mathbf{x}_t + \Delta t \left[-\beta \nabla F(\mathbf{x}_t) - \alpha(t)\mathbf{x}_t + \gamma(\mathbf{x}_t - \mathbf{x}_{t-\Delta t}) \right]. \tag{14}$$

The momentum γ-term helps to overcome the issue of slow convergence due to fluctuations from one iteration to the next, which can cause the state to 'bounce' around an optimum instead of continuously moving towards it. The momentum term reduces the 'bouncing' by pushing consequent updates in the direction of the most recent update.[43] This effect provides a tendency for a system to continue along the path it is already taking, therefore making it less susceptible to 'bouncing' due to fluctuations in the gradient. For example, if a variable equals to 1 at iteration i and is -1 on the iteration $i+1$, then this variable will be pushed at the iteration $i+2$ by the momentum term for a value of 2γ, which provides a good local minima escape

mechansim for the *AOC* algorithm. The parameter γ is generally less than 1 as the dependence of a given state on previous states should weaken with the number of iterations between the current state and the earlier state.

The physical interpretation for the momentum method equation 13 is well-known: in terms of continuous dynamical system, the momentum parameter is equivalent to the point mass m of Newtonian particles moving in viscous medium with friction coefficient μ under conservative force field $f = \nabla E$ with potential energy $E(x)$:[14]

$$m\frac{d^2\mathbf{x}}{dt^2} + \mu\frac{d\mathbf{x}}{dt} = -\nabla E(\mathbf{x}_t). \tag{15}$$

Similarly, the iterative update rule in equation 14 could be interpreted as the dynamical system of equations:

$$m\frac{d^2\mathbf{x}}{dt^2} + \mu\frac{d\mathbf{x}}{dt} = -\nabla F(\mathbf{x}) - \phi(t)\mathbf{x}, \tag{16}$$

where $\phi(t)$ is the nonconservative force. To find the relation between physical quantities and parameters β, α, and γ, one can discretize equation 16:

$$m\frac{\mathbf{x}_{t+\Delta t} - 2\mathbf{x}_t + \mathbf{x}_{t-\Delta t}}{\Delta t^2} + \mu\frac{\mathbf{x}_{t+\Delta t} - \mathbf{x}_t}{\Delta t} = -\nabla F(\mathbf{x}_t) - \phi(t)\mathbf{x}_t, \tag{17}$$

which can be further rewritten as

$$\mathbf{x}_{t+\Delta t} = \mathbf{x}_t - \frac{\Delta t^2}{m + \mu\Delta t}\nabla F(\mathbf{x}_t) - \frac{\Delta t^2}{m + \mu\Delta t}\phi(t)\mathbf{x}_t + \frac{m}{m + \mu\Delta t}(\mathbf{x}_t - \mathbf{x}_{t-\Delta t}). \tag{18}$$

By comparing terms in equation 14 and equation 18, we get:

$$\beta\Delta t = \frac{\Delta t^2}{m+\mu\Delta t} \tag{19}$$
$$\alpha(t)\Delta t = \frac{\Delta t^2}{m+\mu\Delta t}\phi(t) \tag{20}$$
$$\gamma\Delta t = \frac{m}{m+\mu\Delta t}. \tag{21}$$

Thus, the Newtonian system of equations, described by equation 16, is equivalent to the *AOC* iterative update rule, described by equation 14, with the parameters β representing the scaling factor of the conservative force, $\alpha(t)$ standing for the nonconservative force, and γ corresponding to the particle mass.

The *AOC* algorithm converges to steady states for any positive values β, $\alpha(t)$, and $\gamma \in (0,1)$. These steady states are the minima of the Lyapunov function, representing the energy of the system. Denoting $y = f_{\text{nonlinear}}(x)$ to represent either binary or continuous variables, achieved by applying the elementwise nonlinear function for binary variables and linear function for continuous variables, the Lyapunov function can be written for the *QUMO* objective $F(y)$ for equation 16 as:

$$E = \frac{m}{2}\frac{df^{-1}_{\text{nonlinear}}(\mathbf{y}^T)}{dt}\frac{df^{-1}_{\text{nonlinear}}(\mathbf{y})}{dt} - \frac{1}{2}\mathbf{y}^T W\mathbf{y} - \mathbf{b}^T\mathbf{y} + \phi(t)\sum_{i=1}^{N}\int_0^{y_i} f^{-1}_{\text{nonlinear}}(x)dx. \tag{22}$$

Due to presence of nonconservative force, the overall improving *QUMO* objective value during the time evolution can be worsening for several intermediate consecutive iterations. Towards the end of the time evolution, the annealing term $\phi(t)$ decreases

to zero and the kinetic term vanishes, so the minima of the Lyapunov function correspond to the equilibrium states of the equation 16 and are the minima of the *QUMO* objective function. From the physics perspective, the convergence to the overall better optimal state in the end happens as the energy decreases due to the presence of friction.

We note that the promising results of analog computations have been demonstrated for solving differential equations and performing complex mathematical operations. For example, the Fredholm integral equations of the second kind can be solved using free-space visible radiation,[44] the Hilbert transformation can be performed with a second-order optical integrator,[45] the basic trigonometric operations are enabled with metasurface-based platform,[46] and mathematical operations can be performed using high-index acoustic metamaterials.[47]

G.11 Comparison of the *AOC* approach to other iterative approaches

In case of the *QUMO* objective function, the simulated iterative update rule of the *AOC* algorithm reads as:

$$\mathbf{x}_{t+1} = \mathbf{x}_t + \Delta t \left[\alpha(t)(W f_{\text{nonlinear}}(\mathbf{x}_t) + \mathbf{b}) - \beta(t)\mathbf{x}_t + \gamma(\mathbf{x}_t - \mathbf{x}_{t-1})\right], \tag{23}$$

$$f_{\text{nonlinear}}([\mathbf{x}_t]_i) = \begin{cases} \text{sign}([\mathbf{x}_t]_i), & \text{if } [\mathbf{x}_t]_i \text{is constrained to be binary} \\ [\mathbf{x}_t]_i, & \text{if } [\mathbf{x}_t]_i \text{is continuous,} \end{cases}$$

where each variable $[\mathbf{x}_t]_i$ is also clipped to the range of $[-1, 1]$. For the well-studied *QUBO* model, the *AOC* algorithm can be further rewritten in a simpler form as:

$$\mathbf{x}_{t+1} = \mathbf{x}_t + \Delta t \left[\alpha(t)(W \cdot \text{sign}(\mathbf{x}_t) + \mathbf{b}) - \beta(t)\mathbf{x}_t + \gamma(\mathbf{x}_t - \mathbf{x}_{t-1})\right]. \tag{24}$$

where all variables are now assumed to be binary. We choose sign nonlinearity here, but other nonlinearities could be considered including $\{\cos, \tanh, clamp\}$. As we discussed above in Sec. G.10, the *AOC* iterative update rule corresponds to the second order differential equation. Consequently, the *AOC* algorithm is distinct from all the first order methods, the well-known example of which is the Hopfield networks.[48,49] The Euler update rule and ordinary differential equation can be written for Hopfield networks as:

$$\mathbf{x}_{t+1} = \mathbf{x}_t + \Delta t \left[\alpha(t)(W \cdot \tanh(\mathbf{x}_t) + \mathbf{b}) - \beta \mathbf{x}_t\right], \tag{25}$$

$$\frac{d\mathbf{x}}{dt} = \alpha(W \cdot \tanh(\mathbf{x}) + \mathbf{b}) - \beta \mathbf{x}. \tag{26}$$

In these equations, the losses term is represented by a constant parameter β. By considering time-varying losses $\beta(t)$ in Hopfield networks, one arrives to the equations, that are fundamentally similar to our current opto-electronic *AOC* implementation.

Another recently introduced heuristic method is called the simulated bifurcation

algorithm[17,50] which is described by equations:

$$\frac{d\mathbf{x}}{dt} = a_0\mathbf{y}, \tag{27}$$

$$\frac{d\mathbf{y}}{dt} = c_0 Q \cdot sign(\mathbf{x}) - (a_0 - a(t))\mathbf{x}, \tag{28}$$

where a_0, c_0, and $a(t)$ are the hyperparameters. This system of equations can be further reduced to the single second order differential equation:

$$\frac{d^2x}{dt^2} = c_0 Q \cdot sign(x) - (a_0 - a(t))x. \tag{29}$$

Compared to the *AOC* algorithm equations, the simulated bifurcation method doesn't have the first order derivative and its iterative update rule can be written as:

$$\mathbf{x}_{t+1} = \mathbf{x}_t + (\mathbf{x}_t - \mathbf{x}_{t-1}) + \Delta t^2 \left[-(a_0 - a(t))\mathbf{x}_t + c_0 Q \cdot sign(\mathbf{x}_t)\right]. \tag{30}$$

This update rule is similar to the *AOC* algorithm with $\gamma = 1$. In general, the momentum methods with $\gamma \geq 1$ are unstable.[51] Such instability is probably mitigated in the simulated bifurcation algorithm by an extra condition that manually forces the difference $(\mathbf{x}_t - \mathbf{x}_{t-1})$ to be zero if $|\mathbf{x}_t| = 1$. We also note that the original iterative update rule of the simulated bifurcation algorithm uses symplectic Euler method for discretising the system of equations above and can be written as:

$$\mathbf{x}_{t+1} = \mathbf{x}_t + a_0\Delta t \left[(\mathbf{x}_t - \mathbf{x}_{t-1}) - \Delta t(a_0 - a(t))\mathbf{x}_t + \Delta t c_0 Q \cdot sign(\mathbf{x}_t)\right]. \tag{31}$$

which would suffer from the same instability issue due to the momentum term without applying the extra constraint above.

References

[1] A. S. Sotirova et al. "Low cross-talk optical addressing of trapped-ion qubits using a novel integrated photonic chip". *Light: Science & Applications* 13.1 (2024).

[2] K. Weber et al. "Positional Accuracy of 3D Printed Quantum Emitter Fiber Couplers". *Advanced Quantum Technologies* 7.11 (2024).

[3] G. Van der Plas and B. Verbruggen. "A 150MS/s 133μ W 7b ADC in 90nm digital CMOS Using a Comparator-Based Asynchronous Binary-Search sub-ADC". *2008 IEEE International Solid-State Circuits Conference - Digest of Technical Papers*. IEEE, 2008.

[4] C. Kromer et al. "A low-power 20-GHz 52-dB/spl Omega/ transimpedance amplifier in 80-nm CMOS". *IEEE Journal of Solid-State Circuits* 39.6 (2004).

[5] T. Wang et al. "An optical neural network using less than 1 photon per multiplication". *Nature Communications* 13.1 (2022).

[6] J. W. Goodman et al. "Application Of Optical Communication Technology To Optical Information Processing". *Los Alamos Conference on Optics 1979*. Ed. by D. H. Liebenberg. Vol. 0190. SPIE, 1980.

[7] D. Psaltis and N. Farhat. "Optical information processing based on an associative-memory model of neural nets with thresholding and feedback". *Optics Letters* 10.2 (1985).

[8] N. H. Farhat et al. “Optical implementation of the Hopfield model”. *Applied Optics* 24.10 (1985).

[9] Y. Zhang et al. “Graphene oxide for nonlinear integrated photonics”. *Laser & Photonics Reviews* 17.3 (2023).

[10] K. Takata et al. “A 16-bit coherent Ising machine for one-dimensional ring and cubic graph problems”. *Scientific reports* 6.1 (2016).

[11] C. Anil et al. *Path Independent Equilibrium Models Can Better Exploit Test-Time Computation*. 2022. arXiv: 2211.09961 [cs.LG].

[12] Y. Du et al. “Learning iterative reasoning through energy minimization”. *International Conference on Machine Learning*. PMLR. 2022.

[13] D. Georgiev, P. Liò, and D. Buffelli. *The Deep Equilibrium Algorithmic Reasoner*. 2024. arXiv: 2402.06445 [cs.LG].

[14] N. Qian. “On the momentum term in gradient descent learning algorithms”. *Neural Networks* 12.1 (1999).

[15] S. Reifenstein et al. “Coherent ising machines with optical error correction circuits”. *Advanced Quantum Technologies* 4.11 (2021).

[16] S. Kirkpatrick and D. Sherrington. “Solvable model of a spin-glass”. *Phys. Rev. Lett* 35.26 (1975).

[17] H. Goto et al. “High-performance combinatorial optimization based on classical mechanics”. *Science Advances* 7.6 (2021).

[18] L. Braine et al. “Quantum Algorithms for Mixed Binary Optimization Applied to Transaction Settlement”. *IEEE Transactions on Quantum Engineering* 2 (2021).

[19] F. Furini et al. “QPLIB: a library of quadratic programming instances”. *Mathematical Programming Computation* 11 (2 2019).

[20] F. Furini and E. Traversi. *QPLIB: A Library of Quadratic Programming Instances*. Zuse Institute Berlin. 2021. URL: http://qplib.zib.de/ (visited on 01/20/2023).

[21] *G-set instances*. URL: https://web.stanford.edu/~yyye/yyye/Gset/.

[22] D. Perera et al. “Chook–A comprehensive suite for generating binary optimization problems with planted solutions”. *arXiv preprint arXiv:2005.14344* (2020).

[23] *Interactive charts comparing the results of Hans Mittelmann’s benchmarks.* 2023. URL: https://mattmilten.github.io/mittelmann-plots/.

[24] M. Aramon et al. “Physics-inspired optimization for quadratic unconstrained problems using a digital annealer”. *Frontiers in Physics* 7 (2019).

[25] M. Mohseni et al. *Nonequilibrium Monte Carlo for unfreezing variables in hard combinatorial optimization*. 2021. arXiv: 2111.13628 [cond-mat.dis-nn].

[26] B. Tasseff et al. *On the Emerging Potential of Quantum Annealing Hardware for Combinatorial Optimization*. 2022. arXiv: 2210.04291 [math.OC].

[27] *Microsoft Quantum Inspired Optimisation (QIO) provider*. 2022. URL: https://web.archive.org/web/20230331171629/https://learn.microsoft.com/en-us/azure/quantum/provider-microsoft-qio.

[28] J. N. Hooker and M. A. Osorio. "Mixed logical-linear programming". *Discrete Applied Mathematics* 96 (1999).

[29] T. Achterberg et al. "Presolve reductions in mixed integer programming". *INFORMS Journal on Computing* 32.2 (2020).

[30] E. Marinari and G. Parisi. "Simulated tempering: a new Monte Carlo scheme". *Europhysics letters* 19.6 (1992).

[31] *Gurobi optimizer reference manual*. 2023. URL: http://www.gurobi.com.

[32] F. Hamze et al. "Wishart planted ensemble: A tunably rugged pairwise Ising model with a first-order phase transition". *Physical Review E* 101.5 (2020).

[33] F. Hamze. "Rotationally-Constrained Discrepancy Problems (RCDPs)" (2023).

[34] F. Hamze et al. "From near to eternity: spin-glass planting, tiling puzzles, and constraint-satisfaction problems". *Physical Review E* 97.4 (2018).

[35] C. Helmberg and F. Rendl. *A Spectral Bundle Method for Semidefinite Programming*. report SC 97-37. Konrad-Zuse-Zentrum für Informationstechnik Berlin, 1997.

[36] N. Mohseni, P. L. McMahon, and T. Byrnes. "Ising machines as hardware solvers of combinatorial optimization problems". *Nature Reviews Physics* 4.6 (2022).

[37] R. Brown et al. *Copositive programming for mixed-binary quadratic optimization via Ising solvers*. 2022. arXiv: 2207.13630 [math.OC].

[38] *Fujitsu Digital Annealer*. 2021. URL: https://www.fujitsu.com/global/documents/about/research/techintro/3rd-g-da_en.pdf.

[39] M. P. Harrigan et al. "Quantum approximate optimization of non-planar graph problems on a planar superconducting processor". *Nature Physics* 17.3 (2021).

[40] Y. R. Sanders et al. "Compilation of fault-tolerant quantum heuristics for combinatorial optimization". *PRX Quantum* 1.2 (2020).

[41] *D-Wave machine*. 2023. URL: https://www.dwavesys.com/.

[42] B. T. Polyak. "Some methods of speeding up the convergence of iteration methods". Trans. from the Russian by H. P. Cleaves. *USSR Computational Mathematics and Mathematical Physics* (1962).

[43] D. E. Rumelhart, G. E. Hinton, and R. J. Williams. "Learning representations by back-propagating errors". *Nature* 323.6088 (1986).

[44] A. Cordaro et al. "Solving integral equations in free space with inverse-designed ultrathin optical metagratings". *Nature Nanotechnology* (2023).

[45] R. Ashrafi et al. "Time-delay to intensity mapping based on a second-order optical integrator: application to optical arbitrary waveform generation". *Optics Express* 23.12 (2015).

[46] Z. Zhao et al. "Deep learning-enabled compact optical trigonometric operator with metasurface". *PhotoniX* 3.1 (2022).

[47] F. Zangeneh-Nejad and R. Fleury. “Performing mathematical operations using high-index acoustic metamaterials”. *New Journal of Physics* 20.7 (2018).

[48] J. J. Hopfield. “Neural networks and physical systems with emergent collective computational abilities.” *Proceedings of the National Academy of Sciences* 79.8 (1982). eprint: https://www.pnas.org/doi/pdf/10.1073/pnas.79.8.2554.

[49] J. J. Hopfield and D. W. Tank. “‘Neural’ computation of decisions in optimization problems”. *Biological Cybernetics* 52.3 (1985).

[50] H. Goto, K. Tatsumura, and A. R. Dixon. “Combinatorial optimization by simulating adiabatic bifurcations in nonlinear Hamiltonian systems”. *Science Advances* 5.4 (2019). eprint: https://advances.sciencemag.org/content/5/4/eaav2372.full.pdf.

[51] M. Torii and M. T. Hagan. “Stability of steepest descent with momentum for quadratic functions”. *IEEE Transactions on Neural Networks* 13.3 (2002).
